# Roles of the m^6^A Modification of RNA in the Glioblastoma Microenvironment as Revealed by Single-Cell Analyses

**DOI:** 10.3389/fimmu.2022.798583

**Published:** 2022-04-26

**Authors:** Feng Yuan, Xiangming Cai, Zixiang Cong, Yingshuai Wang, Yuanming Geng, Yiliyaer Aili, Chaonan Du, Junhao Zhu, Jin Yang, Chao Tang, Aifeng Zhang, Sheng Zhao, Chiyuan Ma

**Affiliations:** ^1^Department of Neurosurgery, Affiliated Jinling Hospital, Medical School of Nanjing University, Nanjing, China; ^2^School of Medicine, Southeast University, Nanjing, China; ^3^Department of Internal Medicine III, University Hospital Munich, Ludwig Maximilians-University Munich, Munich, Germany; ^4^Department of Neurosurgery, The Affiliated Jinling Hospital of Nanjing Medical University, Nanjing, China; ^5^Department of Neurosurgery, Jinling Hospital, Nanjing, China; ^6^Department of Pathology, School of Medicine, Southeast University, Nanjing, China; ^7^Department of Biochemistry and Molecular Biology, School of Medicine, Southeast University, Nanjing, China; ^8^The Key Laboratory of Developmental Genes and Human Disease, Institute of Life Sciences, Southeast University, Nanjing, China; ^9^Department of Neurosurgery, Jinling Hospital, The First School of Clinical Medicine, Southern Medical University, Nanjing, China

**Keywords:** glioblastoma, immune microenvironment, m^6^A, single-cell analysis, cell communication

## Abstract

**Purpose:**

Glioblastoma multiforme (GBM) is a common and aggressive form of brain tumor. The N^6^-methyladenosine (m^6^A) mRNA modification plays multiple roles in many biological processes and disease states. However, the relationship between m^6^A modifications and the tumor microenvironment in GBM remains unclear, especially at the single-cell level.

**Experimental Design:**

Single-cell and bulk RNA-sequencing data were acquired from the GEO and TCGA databases, respectively. We used bioinformatics and statistical tools to analyze associations between m^6^A regulators and multiple factors.

**Results:**

*HNRNPA2B1* and *HNRNPC* were extensively expressed in the GBM microenvironment. m^6^A regulators promoted the stemness state in GBM cancer cells. Immune-related BP terms were enriched in modules of m^6^A-related genes. Cell communication analysis identified genes in the GALECTIN signaling network in GBM samples, and expression of these genes (*LGALS9*, *CD44*, *CD45*, and *HAVCR2*) correlated with that of m^6^A regulators. Validation experiments revealed that *MDK* in MK signaling network promoted migration and immunosuppressive polarization of macrophage. Expression of m^6^A regulators correlated with ICPs in GBM cancer cells, M2 macrophages and T/NK cells. Bulk RNA-seq analysis identified two expression patterns (low m^6^A/high ICP and high m^6^A/low ICP) with different predicted immune infiltration and responses to ICP inhibitors. A predictive nomogram model to distinguish these 2 clusters was constructed and validated with excellent performance.

**Conclusion:**

At the single-cell level, m^6^A modification facilitates the stemness state in GBM cancer cells and promotes an immunosuppressive microenvironment through ICPs and the GALECTIN signaling pathway network. And we also identified two m^6^A-ICP expression patterns. These findings could lead to novel treatment strategies for GBM patients.

## Introduction

Glioblastoma multiforme (GBM) is the most common primary tumor of the central nervous system, and it is extremely aggressive (1). Standard treatment for GBM is surgical resection followed by chemoradiotherapy, with a 5-year survival of 7.2% (1, 2). Immunotherapy has achieved growing success across systemic cancers, and has become a prominent player in the treatment of GBM (3). Nonetheless, the high degree of genetic heterogeneity and immunosuppressive microenvironment that characterize GBM represent important challenges to the application of immunotherapy to this disease (1). Advancements in knowledge of the immune cells in the GBM microenvironment, particularly glioma-associated microglia, macrophages and T cells, might lead to novel strategies to strengthen anti-tumor immunity (3).

An important aspect of the behavior of these key immune cells involves the N^6^-methyladenosine (m^6^A) modification, which has emerged as the most abundant chemical modification of protein-coding and noncoding RNAs (4, 5). To date, regulators of the m^6^A modification have been reported to be involved in cancer biology, including cancer progression and other processes (4). Notably, the roles of m^6^A regulators in mediating immunotherapy resistance have been highlighted in recent studies. For instance, expression of *METTL3* in macrophages was suggested to synergize with PD-1-based therapy in B16 melanoma (6), and *METTL3* and *METTL14* have been shown to regulate immune response to anti-PD-1 treatment in melanoma and colorectal carcinoma (7). Together, these findings indicate a role for m^6^A regulators as potential therapeutic targets in anticancer immunotherapy.

Several m^6^A regulators have been reported to be upregulated and to play vital roles in GBM, including enhancing cell self-renewal and proliferation in glioblastoma stem cells (GSCs) (8). However, some opposing findings have shown lower expression levels of some m^6^A writers in GBM and have detected anticancer properties of m^6^A regulators (8, 9). This conflict suggests a complex role of m^6^A-related methylation in the occurrence and development of GBM. Notably, though, *FTO*, *YTHDF2*, and *RBM15* were found to have prognosis predictive value in GBM (8, 10). In addition, patients with higher m^6^A related risk scores were more sensitive to temozolomide treatment and showed lower drug resistance overall (10).

**Figure 1:**
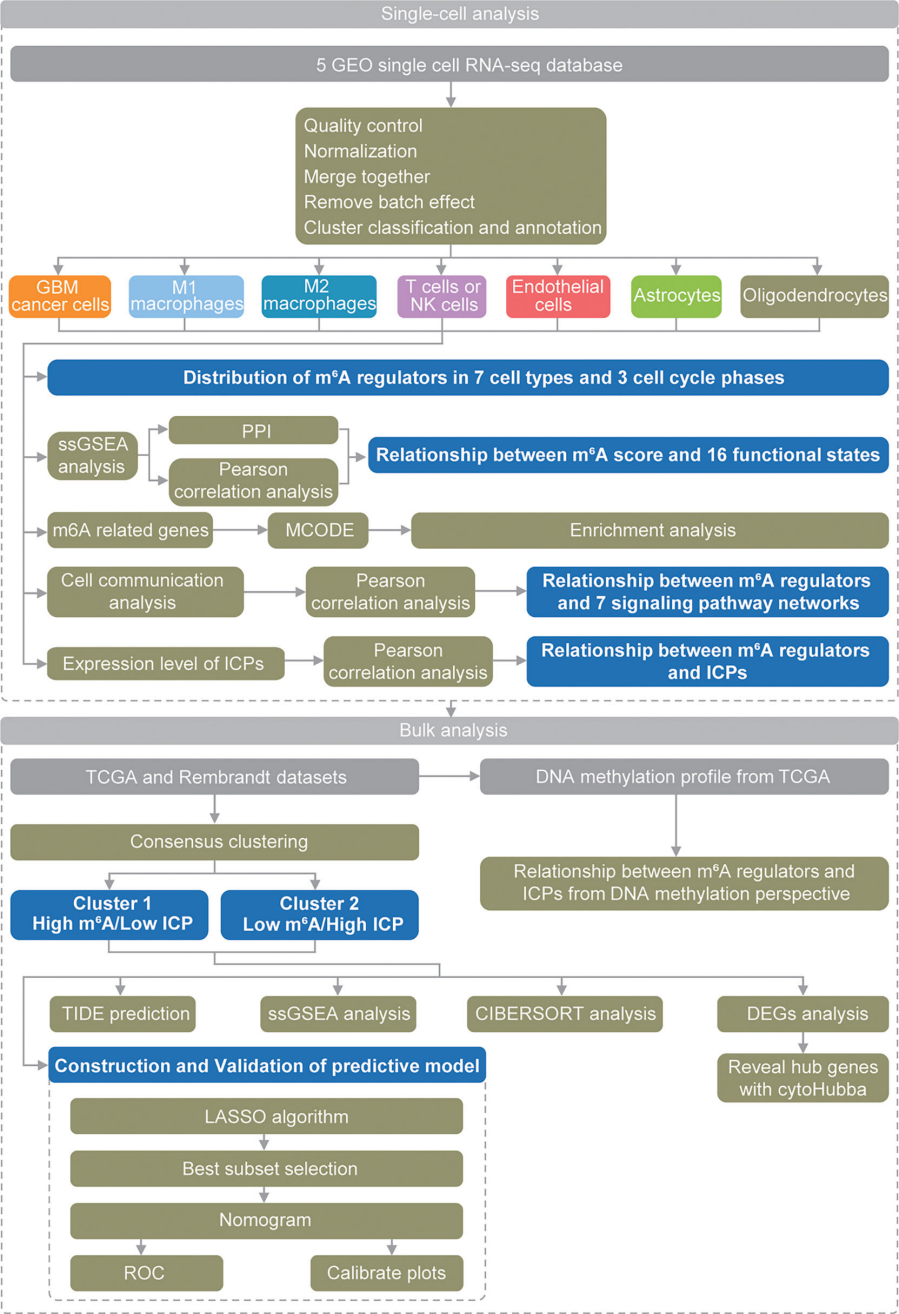
Schematic diagram of the study design.

These findings suggested critical and complex roles of m^6^A modification in GBM, and more research opportunities and challenges have emerged. Recent development of single-cell analysis methods provides more comprehensive approaches to explore the potential mechanism of action of m^6^A modifications in GBM at the cellular level. Herein, for the first time, we used scRNA-seq data to thoroughly analyze (**Figure 1**) the roles of m^6^A modifications in the GBM microenvironment, especially its relationship with functional states, cell communication and immune checkpoints (ICPs).

## Materials and Methods

### Datasets

Five publicly available human GBM scRNA-seq datasets (11–15) were obtained from the Gene Expression Omnibus (GEO) database (http://www.ncbi.nlm.nih.gov/geo/). Bulk RNA-seq datasets and DNA methylation profiles were obtained from the Cancer Genome Atlas (TCGA, https://portal.gdc.cancer.gov/) and the Repository of Molecular Brain Neoplasia Data (REMBRANDT) (16) datasets. These bulk datasets were downloaded *via* the University of California, Santa Cruz (UCSC) Xena browser (https://xenabrowser.net/) and Chinese Glioma Genome Atlas (CGGA, http://www.cgga.org.cn/) websites. A summary description of included scRNA-seq datasets is provided in **Supplementary Table S2**.

### Single-Cell RNA-seq Analysis

The “Seurat” R package (version 4.0.2) (17) was used to perform single-cell RNA-seq analyses. We only included in this study samples with at least 10 000 detected genes. Quality control (QC) was based on following standards: 1) genes detected in fewer than 3 cells were excluded; 2) cells with fewer than 200 total detected genes were excluded; 3) cells with at least 5% (for GSE141383, GSE84465, GSE103224, GSE89567) or 10% (for GSE138794) of mitochondrial genes were excluded; 4) cells with at most 3% (for GSE141383, GSE138794, GSE103224, GSE89567) or 1% (for GSE84465) of ribosomal genes were excluded; 5) cells with at least 0.1% of hemoglobin genes were excluded and 6) cells with total gene expression between first quartile − 1.5 × interquartile range (IQR) and third quartile + 1.5 × IQR were retained to exclude cellular doublets.

Unnormalized datasets were normalized with the “NormalizeData” function in R. Then, these five datasets were merged together, and the zero imputation method was applied to investigate missing data relating to significant markers (**Supplementary Table S3**). The batch effect was removed *via* the “fastMNN” algorithm from the “SeuratWrappers” R package (version 0.3.0).

Principal component analysis (PCA) was performed, and the top 20 PCs were used for cluster classification. The initial 20 PCs were also utilized in the further visualization process through the t-distributed stochastic neighbor embedding (t-SNE) algorithm. After cluster classification, different cell clusters were identified and annotated manually within the CellMarker database (http://biocc.hrbmu.edu.cn/CellMarker/) (18). The cell cycle score was calculated with the “CellCycleScoring” function. Cell communication network analysis was conducted with the “CellChat” R package (version 1.1.1). Gene Expression Profiling Interactive Analysis (GEPIA) (http://gepia.cancer-pku.cn/) was used to compare the expression levels of m^6^A regulators between GBM and normal samples with bulk RNA-seq datasets.

### Single-Sample Gene Set Enrichment Analysis

The ssGSEA algorithm was conducted with the “GSVA” R package (version 1.34.0) to calculate enrichment scores for several gene signatures. The gene set for m^6^A included 13 m^6^A readers (*YTHDC1*, *YTHDC2*, *IGF2BP1*, *IGF2BP2*, *IGF2BP3*, *YTHDF1*, *YTHDF2*, *YTHDF3*, *HNRNPA2B1*, *HNRNPC*, *ELAVL1*, *LRPPRC* and *FMR1*), 8 m^6^A writers (*METTL3*, *METTL14*, *WTAP*, *KIAA1429*, *RBM15*, *RBM15B*, *ZC3H13* and *CBLL1*) and 2 m^6^A erasers (*FTO* and *ALKBH5*). The gene lists (**Supplementary Table S1**) for other biological states were from CancerSEA (http://biocc.hrbmu.edu.cn/CancerSEA).

### m^6^A Related Genes

Analyses of Pearson correlations between m^6^A scores and gene expression were conducted to filter m^6^A related genes under the following criteria: (1) R value greater than 0.3 or less than -0.3 and (2) *P* < 0.05. In M2 macrophages, we strengthened the criteria for m^6^A related genes to R value greater than 0.5 or less than -0.5. The Molecular Complex Detection (MCODE) plugin from Cytoscape software (version 3.8.1) was applied to identify core gene modules with scores greater than 10.

### Functional Annotation

Enrichment analyses of biological processes (BP) Gene Ontology (GO) annotations and Kyoto Encyclopedia of Genes and Genomes (KEGG) pathway analyses were performed based on the “clusterProfiler” R package (version 3.14.3) with adjusted *P* < 0.05 as the cutoff criterion.

### Protein-Protein Interaction Network Analysis

The PPI network was constructed with Search Tool for the Retrieval of Interacting Genes (STRING) (https://string-db.org/) and visualized with Cytoscape.

### Bulk RNA-seq Analysis

To better understand the interaction between m^6^A regulators and immune checkpoints (ICPs), the “ConsensusClusterPlus” R package (version 1.50.0) was applied to identify distinct clusters of bulk RNA-seq samples with correlated expression levels of m^6^A regulators and ICPs. The Tumor Immune Dysfunction and Exclusion (TIDE) algorithm was utilized to predict potential therapeutic responses of ICP inhibitors. Morpheus (https://software.broadinstitute.org/morpheus) was used to identify differentially expressed genes (DEGs) between clusters. The cutoff criteria for DEGs were: *P* < 0.05 and |log2 fold-change| ≥ 1. An enrichment analysis of DEGs was conducted and hub genes in DEGs were visualized in a PPI network. The ssGSEA for various gene signatures was performed for each sample. We also conducted the Cell Type Identification by Estimating Relative Subsets of RNA Transcripts (CIBERSORT) analysis of the abundance scores of immune cells in GBM samples. A Pearson correlation analysis among the methylation levels and expression levels of m^6^A regulators and ICPs was performed and visualized with Cytoscape software.

### Construction of Predictive Model

To sort GBM patients into 2 clusters, the TCGA and REMBRANDT datasets were used as a training dataset and validation dataset separately. A least absolute shrinkage and selection operator (LASSO) regression analysis was used to identify all genes with *P* < 0.05. Then, best subset selection was performed to determine the final multivariate logistic regression model. A nomogram was constructed to show the predictive model. The evaluation of the nomogram model was performed with receiver operating characteristic (ROC) curve analysis and calibration curve analysis. “glmnet” (version 4.1), “rms” (version 6.1.0), and “timeROC” (version 0.4) R packages were used for the construction of predictive model.

### Sample Source

Tumor tissues were surgically harvested at Jinling Hospital in accordance with institution‐approved protocols. The tissues were collected from 2 glioma patients (without preoperative radiotherapy or chemotherapy), confirmed pathologically as specimens of grade IV gliomas, and fixed in formalin before paraffin-embedded. Tissues from two nontumoral cases were also collected as control. Written informed consent had been obtained from participants.

### Cell Lines and Culture

The human GBM cell line U87MG were purchased from cell bank of Shanghai Institute of Biochemistry and Cell Biology and grown in Dulbecco’s Modified Eagle’s medium (DMEM) containing 10% fetal bovine serum (FBS, Gibco, USA). THP-1 was kindly provided by Wentao Liu laboratory, Nanjing Medical University and cultured in 1640 medium with 10% FBS. Cells were cultured at 37°C in a humidified atmosphere with 5% CO2.

### Proteins and Reagents

Antibodies against *MDK* (11009-1-AP) and *CD206* (60143-1-lg) was purchased from proteintech, and that against *LRP1* (ab92544) was from Abcam. The β-Actin polyclonal antibody (AP0060) and goat anti-rabbit IgG-HRP (BS13278) were purchased from Bioworld. Midkine protein (abs00930) was from Absin Bioscience Inc. The jetPRIME transfection reagent (101000046) was from Polyplus. Anti-CD206 APC (17–2069–41) and its isotype control (17–4714–81) were purchased from eBioscience. And anti-CD11b FITC (101205), its isotype control (400633), and cell staining buffer (420201) was purchased from BioLegend.

### Transfections With siRNA

The MDK small interfering RNA (siMDK; forward, 5’-GACCA AAGCA AAGGC CAAATT-3’; reverse, 5’-UUUGG CCUUU GCUUU GGUCTT-3’) and negative control siRNA (siCon; forward, 5’-UUCUC CGAAC GUGUC ACGUTT-3’; reverse, 5’-ACGUG ACACG UUCGG AGAATT-3’) were synthesized by GenePharma (Shanghai, China). U87MG was transfected with 100ul/L MDK siRNA and negative control siRNA respectively, using jetPRIME transfection reagent. Cells were transfected with siRNAs for 48 h prior to the following experiments.

### Western Blot Assay

Total proteins of cells were extracted, quantified using BCA kit (23235, Thermo), separated by 10% SDS-PAGE, and transferred onto polyvinylidene difluoride membrane. The membrane was blocked with 5% defatted milk powder for 1.5 h, and was incubated overnight in primary antibody (anti-β-Actin at 1:10000; anti-MDK at 1:1000) at 4°C. On the following day, the membrane was incubated in secondary antibody (goat anti-rabbit IgG-HRP 1:20000) for 1 h at room temperature. After adding ECL chromogenic substrate (Millipore, US), the membrane was imaged using a gel imaging system.

### qPCR

RNA was extracted using Ultrapure RNA Kit (CW0597, CWBIO). cDNA was synthesized using a reverse transcription kit (AE311, TransGern) following the instructions in the manual. The endogenous levels of MDK mRNA were determined using the SYBRGreen PCR Kit (AQ131, TransGern). PCR conditions were: denaturation at 94°C for 30 s, followed by 40 cycles of denaturation at 94°C for 5 s, annealing at 61°C for 35 s, followed by elongation at 95°C for 10 s. GAPDH was used as reference gene for MDK. The following primers were used: MDK-F (5’-AAGGATTGCGGCGTGGGTTTC-3’), MDK-R (5’-TGGCGGACTTTGGTGCCTGTG-3’), GAPDH-F (5’-AATCCCATCACCATCTTCCA-3’), and GAPDH-R (5’-AAATGAGCCCCAGCCTTCT-3’). Relative expression level of MDK was calculated using 2-▵▵Ct approach.

### Transwell Assay for Cell Migration

THP-1 was primed with 185 ng/ml PMA for 24 hours, and then added to the top chamber of Transwell unit at a density of 3 × 10^5^ cells/unit. Next, U87MG cell suspension was added into the lower chamber. The transwell chamber was cultured at 37°C for 24 h in a cell incubator. The cells were fixed with methanol for 15 min, and stained with 0.1% crystal violet solution for 5 min. Then, cells on the upper surface of the chamber bottom were removed gently with a cotton swab. Finally, observe the invaded cells on the lower surface under an inverted microscope. Three separate membranes were analyzed for each condition.

### Flow Cytometry

Staining for cell surface markers was done by re-suspending each sample in 100 µl cell staining buffer containing the antibody cocktail. Three groups were set here. Isotype controls were added into group 1. Anti-CD206 APC and anti-CD11b FITC were added into group 2 (siCon) and 3 (siMDK). Cells were incubated at 4°C for 30 minutes and then washed with cell staining buffer. Cells with CD11b^+^/CD206^+^ phenotype were identified as M2 macrophages. Data were immediately acquired using CytoFLEX (Beckman Coulter) and analyzed with FlowJo software (version 10.5.3).

### Immunohistochemistry

Paraffin‐embedded clinical tissue specimens were sectioned, dewaxed, and dehydrated. Antigen retrieval was conducted using a pressure cooker for 3 min. Then, sections were washed with 3% methanol H2O2. Subsequently, the sections were incubated overnight at 4°C using primary antibodies against *MDK* (11009-1-AP, 1:500) and *LRP1* (ab92544, 1:300), and treated with biotin-free EnVision detection kit (Dako) for secondary antibody. The labeled antigens were visualized by 3,3’-diaminobenzidine tetrahydrochloride as a chromogen. Finally, the sections were counterstained with hematoxylin. ImageJ software and IHC Profiler plugin were applied for qualitative assessment of IHC slides. Five random fields were observed under microscope.

Sections were scored using Histochemistry score (H-SCORE) method. Staining proportion was scored from 0 to 3, with 0 if negative, 1 if < 25% of cells stained positively, 2 if 26–50% of cells stained positively, and 3 if > 50% of cells stained positively. In addition, the staining intensity was scored as 0, no staining; 1, weak; 2, moderate; and 3, strong. These two scores were multiplied by each other to calculate the expression score of *MDK* and *LRP1*, with 0-3 represents low expression, and 4–9 represents high expression.

### Immunofluorescence Staining

Segments of samples were dehydrated with 15% and 30% sucrose solution sequentially, embedded in OCT compound, and cryosectioned into 8 μm sections. Fixation was conducted with 4% paraformaldehyde for 15 min. After washing with PBS at room temperature, samples were incubated with 0.1% Triton X-100 for 5-15 min, were followed by washing with PBS. Sections were blocked with 10% goat serum for 1 h at room temperature. Primary antibody was added for overnight incubation at 4°C.

On the following day, primary antibody was removed by rinsing with PBS, and TRITC/FITC -labelled secondary antibody (1:100) was added and incubated at room temperature for 30 min and subsequently stained with DAPI (1:400) for 1-2 min. Coverslips were mounted. Images were obtained under a laser scanning confocal microscope.

### Statistical Analysis

All statistical analyses were performed in R software (version 3.6.0 and 4.0.2). Student’s t-test and chi-square test or Fisher’s exact test were used for comparisons of continuous variables and categorical variables. Bar charts were drawn to display the comparisons using the “ggplot” R package (version 3.3.3). The Kaplan–Meier approach was performed to estimate survival, and the overall survival (OS) was compared with log-rank tests. We also applied Pearson correlation analyses to evaluate two continuous variables, after calculating the mean value of these variables in each sample. To avoid the influence of missing data, we excluded samples with missing data in correlation analyses, and only conducted the correlation analysis when there were more than 3 samples. The correlation analysis results were visualized with “corrgram” (version 1.13) and “ggplot” R packages. *P* < 0.05 values were set as indicating statistical significance.

## Results

### Analysis of scRNA-seq Data Identifies 7 Types of Cells in GBM

**Figure 2:**
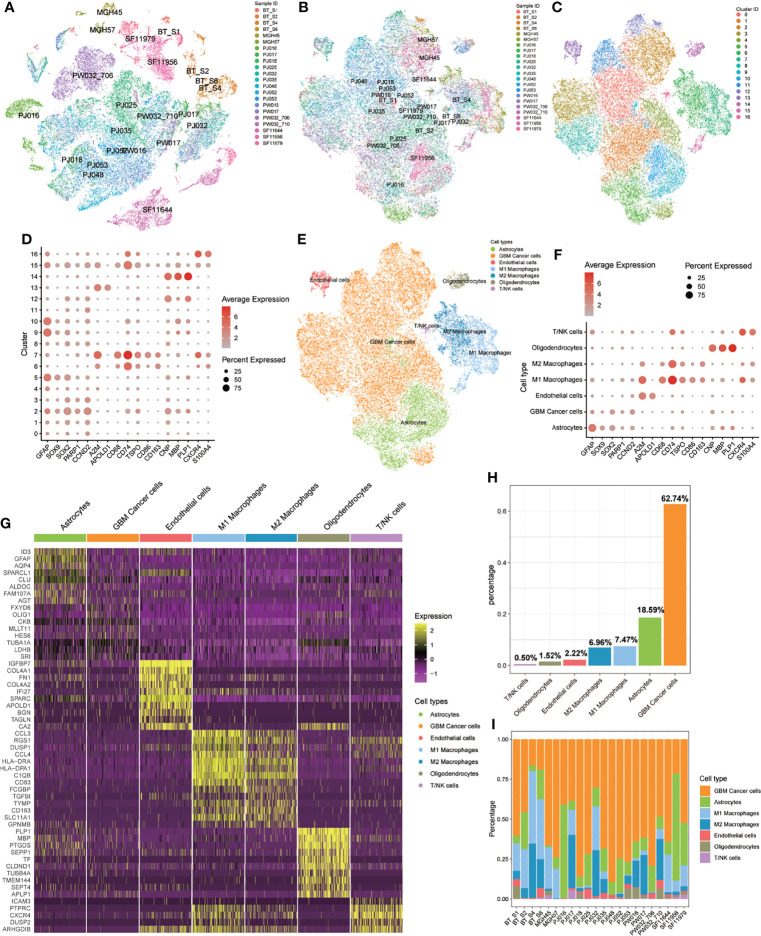
Identification of 17 cell clusters and 7 types of cells in GBM tumors. **(A, B)** tSNE plot of GBM cells before **(A)** and after **(B)** batch effect elimination. **(C)** Unsupervised classification successfully identified 17 cell clusters. **(D)** All 17 clusters were annotated by CellMarker according to the composition of the marker genes. **(E)** tSNE plot of 7 cell types. **(F)** Expression levels of marker genes in the 7 cell types. **(G)** Heatmap of differentially expressed features in each cell type. **(H)** Distribution of 7 cell types for all included cells. **(I)** Distribution of 7 cell types in each included sample.

We obtained scRNA-seq profiles of 52 851 single cells from 22 GBM samples (**Supplementary Table S2**). Following the application of a QC standard, 47 978 single cells were retained (**Supplementary Figure 1**; **Supplementary Table S2**). To visualize the distribution of the scRNA-seq profile, we employed a t-distributed stochastic neighbor embedding (t-SNE) algorithm to reduce the dimensionality of these datasets (**Figure 2A**). After appropriate elimination of the batch effect within these datasets with the “fastMNN” algorithm, the data was well integrated (**Figure 2B**). With unsupervised classification, cells were successfully classified into 17 clusters (**Figure 2C**). Based on the expression patterns of markers from CellMarker, we manually annotated these clusters as the following 7 cell types: 1) GBM cancer cells (expressing *SOX2*, *PARP1*, and *CCND2*); 2) M1 macrophages (*CD68*, *CD74*, *TSPO*, and *CD86*); 3) M2 macrophages (*CD68*, *CD74*, and *CD163*); 4) T cells or natural killer (NK) cells (*CXCR4* and *S100A4*); 5) endothelial cells (*A2M* and *APOLD1*); 6) astrocytes (*GFAP* and *SOX9*); 7) oligodendrocytes (*CNP*, *MBP*, and *PLP1*) (**Figure 2D–F, H**). We also detected heterogeneous cell compositions among these included samples (**Figure 2I**). DEGs for cell types were identified (**Figure 2G**; **Supplementary Table S4**), and enrichment analyses for these DEGs were conducted to show related BP and pathways of each cell type (**Supplementary Tables S4**).

### *HNRNPA2B1* and *HNRNPC* Show Extensive Expression in the GBM Microenvironment

**Figure 3:**
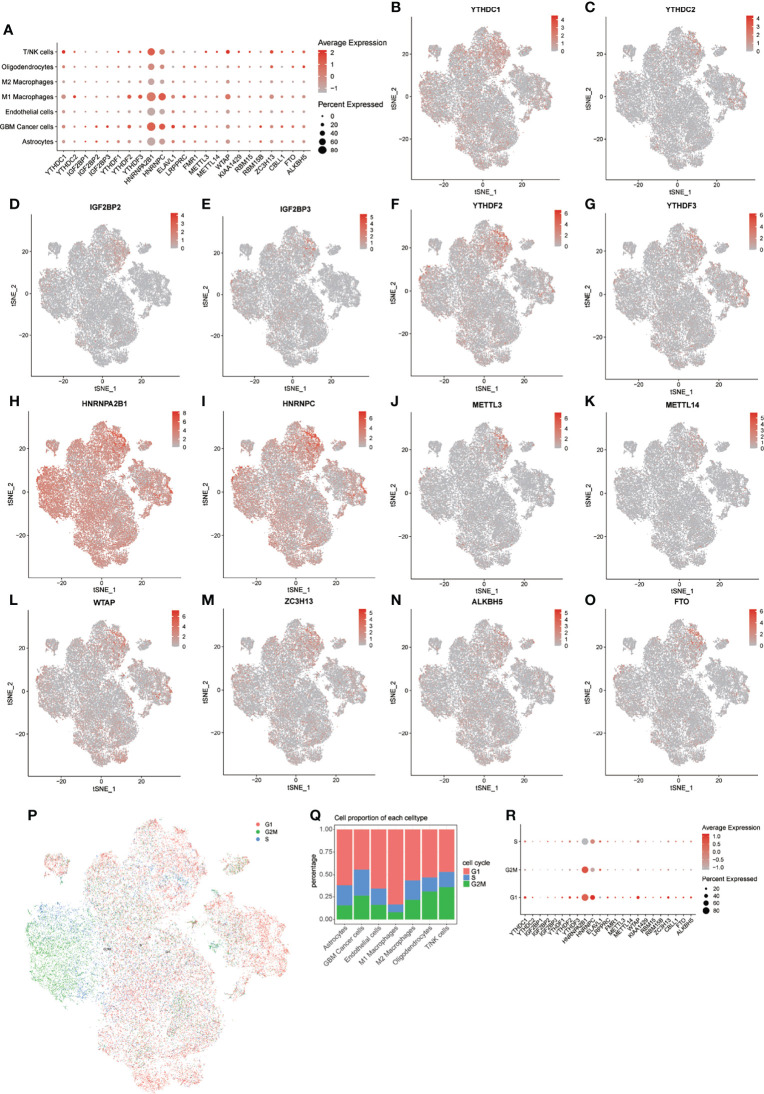
Distribution of m^6^A regulators in GBM microenvironment. **(A)** Expression levels of m^6^A regulators in the 7 cell types. **(B–O)** tSNE plots of 14 m^6^A regulators. **(P)** tSNE plot of cells in 3 cell cycles. **(Q)** Distribution of cells in 3 stages of the cell cycle in 7 cell types. **(R)** Expression levels of m^6^A regulators in 3 stages of the cell cycle.

First, we obtained the expression levels from the bulk transcriptome of 23 m^6^A regulators in GBM and normal samples from GEPIA, and found that most m^6^A regulators have relatively higher expression levels in GBM compared with that in normal samples. Especially notable were *HNRNPA2B1*, *HNRNPC*, and *WTAP* (**Supplementary Figure 2J**). Then, to explore the expression patterns of m^6^A regulators in GBM samples at the single-cell level, we drew t-SNE plots of these m^6^A regulators. In general, all 23 m^6^A regulators were expressed in all 7 cell types, but not in all cells (**Figure 3**; **Supplementary Figures 2A–I**).

Notably, *HNRNPA2B1* and *HNRNPC* showed extensive expression pattern in all cell types, but the expression of these m^6^A regulators was higher in M1 macrophages, GBM cancer cells, and T/NK cells. We further explored the expression levels of these m^6^A regulators in different cell cycle phases (**Figure 3P**). Heterogeneity of cell cycle distribution was observed in these included samples (**Supplementary Figure 3H**). The GBM cancer cells exhibited the most and largest proportion of cells at S and G_2_M phases. Interestingly, other types of cells, except M1 macrophages, had about 30% to 50% cells in the S and G_2_M phases. In general, cells at G_1_ phase showed higher expression of m^6^A regulators than did cells at S and G_2_M phases (**Figure 3R**). For cells at S phase, *HNRNPC* had highest expression level relative to other m^6^A regulators. A higher expression level of *HNRNPA2B1* was observed in cells at G_2_M phase. For cells at G_1_ phase, m^6^A regulators with higher expression levels were *HNRNPA2B1*, *HNRNPC*, *WTAP*, and *YTHDC1*.

Similar results were observed in all 7 types of cells, except for M2 macrophages (**Supplementary Figures 3A–G**). For M2 macrophages at S phase, *HNRNPA2B1* was more highly expressed than was *HNRNPC*. These results indicated intratumor heterogeneity of m^6^A regulators and intertumor heterogeneity of cell types and cell cycle phases in different samples. The following analyses of expression of m^6^A modification factors in GBM were based on subsets of the noted cell types.

### m^6^A Regulators Associate With Functional States

**Figure 4:**
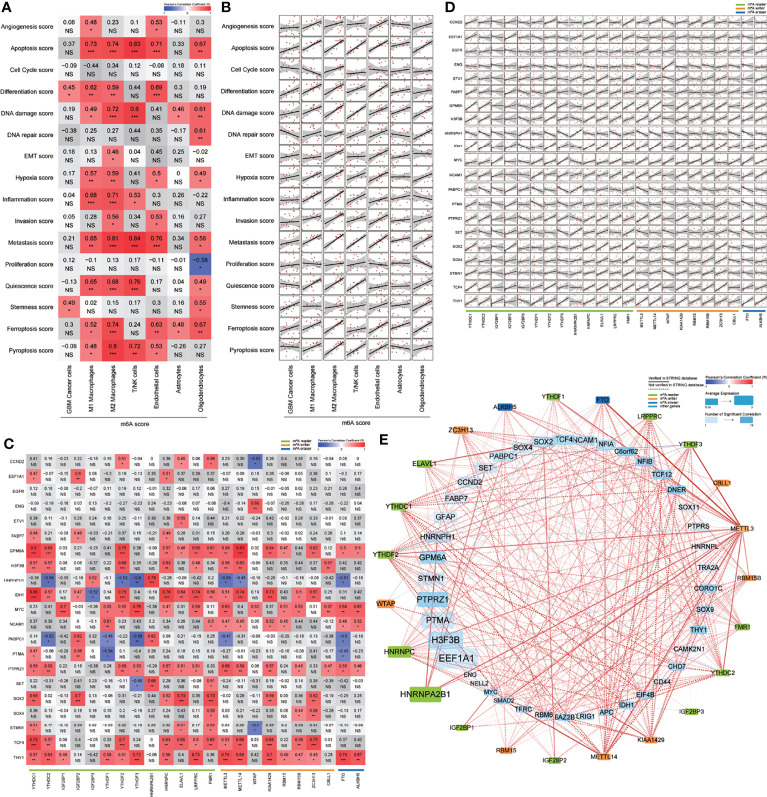
Relationship between m^6^A modification and 16 functional states. **(A, B)** Correlation heatmap **(A)** and correlation analysis **(B)** of m^6^A score and 16 functional state scores in 7 cell types. **(C, D)** Correlation heatmap **(C)** and correlation analysis **(D)** of m^6^A regulators and partial stemness related genes in GBM cancer cells. **(E)** Correlation network of m^6^A regulators and stemness related genes in GBM cancer cells. NS: *P* > 0.05, *P ≤ 0.05, **P ≤ 0.01, ***P ≤ 0.001.

To assess the potential relationship between m^6^A score and scores representing 16 functional states, we conducted a series of Pearson correlation analyses in all 7 cell types (**Figures 4A, B**). Specifically, we found that in GBM cancer cells, the m^6^A score was significantly correlated with the stemness score (R = 0.49, *P* < 0.05). To further explore the relationship between m^6^A regulators and stemness-related genes, we calculated the mean expression levels of genes in each sample, conducted Pearson correlation and PPI analyses between these two sets of genes (**Figures 4C, D**), and visualized these results into an m^6^A regulation network (**Figure 4E**). These results displayed extensive positive correlations between m^6^A regulators and stemness-related genes. Notably, *SOX2*, which can reprogram differentiated glioma cells to glioma stem-like cells (GSCs), has been identified as a *bona fide* m^6^A target of *METTL3 (* 19), but the downstream m^6^A reader of *SOX2* in GBM remains unknown. In colorectal carcinoma, *IGF2BP2* has been proven to be a downstream m^6^A reader of *SOX2 (* 20). Our regulation network suggested that both *IGF2BP2* and *ELAVL1* were potential downstream m^6^A readers of *SOX2* in GBM (**Figure 4E**).

Beside the stemness score in GBM cancer cells, we also found that differentiation score in GBM cancer cells; apoptosis, ferroptosis, and pyroptosis scores in M1 macrophages; epithelial-mesenchymal transition (EMT), invasion, and metastasis scores in M2 macrophages; apoptosis, DNA damage, and pyroptosis scores in T/NK cells; and angiogenesis, invasion, and pyroptosis scores in endothelial cells were all significantly correlated with m^6^A score. We further conducted Pearson correlation analyses between these gene sets and m^6^A regulators. By combining of these correlation analyses with PPI analyses, we constructed 13 m^6^A regulation networks (**Supplementary Figure 4**). These networks provided valuable information about potential regulatory mechanisms of m^6^A regulators in the GBM microenvironment, which will be explored and verified in further studies.

### Modules of m^6^A Related Genes Associate With Immune-Related Terms

By conducting the Pearson correlation analysis between m^6^A score and all genes in all 7 cell types, we filtered m^6^A related genes for each cell type under the *P* < 0.05 criterion. With PPI analysis and the MCODE plugin from Cytoscape software (Degree cutoff = 2; Node score cutoff = 0.2; K-core = 2 and Max depth = 100), we identified functional modules of m^6^A related genes in a PPI network (**Supplementary Figure 5**; **Supplementary Table S5**). Enrichment analysis was applied for each cluster to explore potential related BP and pathways (**Supplementary Table S5**). As expected, all cell types had clusters related to mRNA modification. Also, we found energy conversion and cell cycle related terms in clusters of most cell types. Specifically, terms related to antigen processing and presentation process were enriched in cluster 2 of GBM cancer cells and cluster 1 of endothelial cells (**Supplementary Table S5**). In KEGG analyses, terms relating to “PD-L1 expression and PD-1 checkpoint pathway in cancer” were enriched in cluster 2 of M2 macrophages and cluster 1 and 2 of endothelial cells (**Supplementary Table S5**).

### m^6^A Regulators Promote Immunosuppressive Activities Through the GALECTIN Signaling Network

We performed cell communication analysis in all samples and each sample separately to explore robust signal pathways in the GBM cancer microenvironment. Through subgroup analysis for each included sample, we discovered that genes from the GALECTIN (*LGALS9*, *CD44*, *CD45*, and *HAVCR2*), GRN (*GRN* and *SORT1*), MK (*MDK*, *PTPRZ1*, *NCL*, *ITGA6*, *ITGB1*, *LRP1*, and *SDC4*), PTN (*PTN*, *PTPRZ1*, *NCL*, *SDC3*, and *SDC4*), SPP1 (*SPP1*, *CD44*, *ITGAV*, and *ITGB1*), ANNEXIN (*ANXA1* and *FPR1*), and VISFATIN (*NAMPT* and *INSR*) signaling pathway networks were detected in most samples with consistent network patterns (**Supplementary Figures 6M, N**; **Supplementary Figure 6**; **Supplementary Table S6**). Specifically, M1 and M2 macrophages targeted other cells in the GALECTIN, GRN and SPP1 signaling pathway networks (**Supplementary Figures 6C, D, G, H**), and GBM cancer cells regulated other cells in the MK and PTN signaling pathway network (**Supplementary Figures 6M, N**; **Supplementary Figures 6E, F**).

Next, we conducted a Pearson correlation analysis to investigate whether m^6^A regulators were involved in the regulation of these signaling pathway networks (**Supplementary Figures 7**-**10**). The GALECTIN signaling network plays an important role increasing induced regulatory T (iTreg) cell stability and suppressing T-cell proliferation (21). The expression level of genes in the GALECTIN signaling pathway network were higher in M1 macrophages, M2 macrophages, and T/NK cells (**Supplementary Figure 10C**). Our Pearson correlation analysis showed that in M2 macrophages, expression levels of *YTHDC1*, *YTHDC2*, *IGF2BP2*, *IGF2BP2*, *HNRNPC*, *ELAVL1*, *LRPPRC*, *ZC3H13*, and *ALKBH5* were positively correlated with expression of *LGALS9*, the gene encoding galectin-9 (**Supplementary Figures 8A, B**). In T/NK cells, *CD44* expression was positively correlated with that of *YTHDF2* and *HNRNPA2B1* and negatively correlated with *FTO* and *ALKBH5*. In T/NK cells, expression of *HAVCR2*, which encodes TIM-3, was positively correlated with that of *YTHDF2*. These results suggested that m^6^A modification may promote immunosuppressive activities through the GALECTIN signaling pathway network in the GBM immune microenvironment.

### *MDK* Induces Macrophage Migration and M2 Polarization

**Figure 5:**
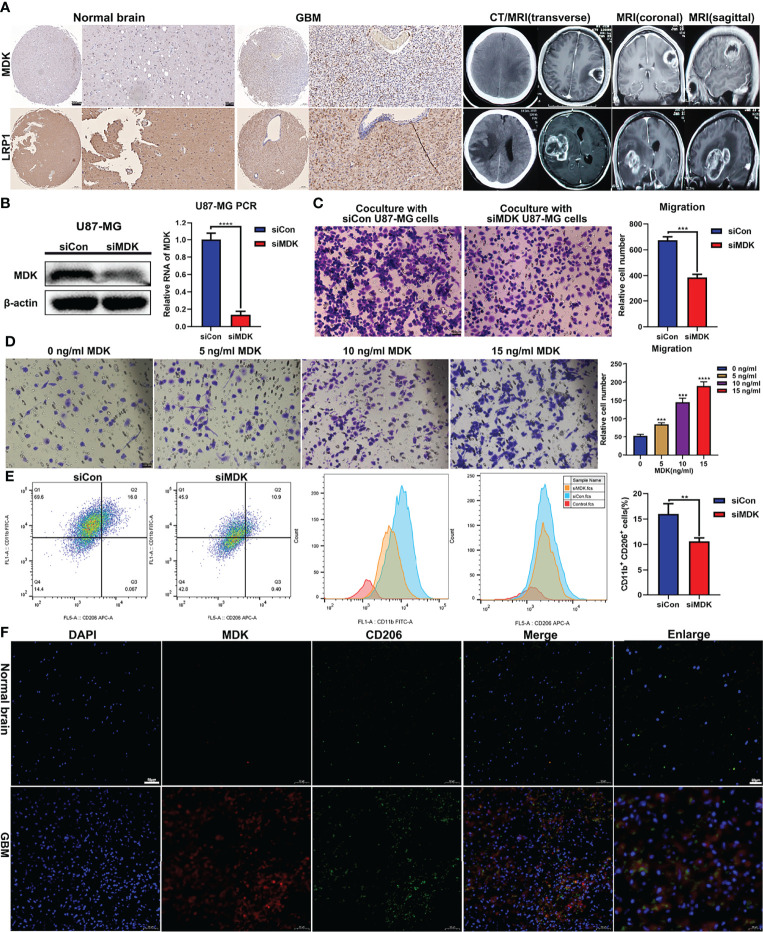
*MDK* promotes migration and immunosuppressive polarization of macrophage. **(A)** The expression of *MDK* and *LRP1* in GBM tissues (n = 2) and normal tissues (n = 2) determined using IHC (scale bar = 50 μm). And the CT and MRI images of GBM patients. **(B)** The siRNA knockdown effect confirmed with western blot and qPCR experiments. **(C)** THP-1 was treated with 185 ng/ml PMA for 24 h in the upper chamber of transwell unit. And then, coculture it with supernate from U87MG medium in the lower chamber for 24 h. The image showed the macrophages migrate through the membrane of chamber (scale bar = 100 μm). **(D)** THP-1 was treated with 185 ng/ml PMA for 24 h in the upper chamber of transwell unit. And then, coculture it with MDK protein at various concentration gradients in the lower chamber for 24 h. The image showed the macrophages migrate through the membrane of chamber (scale bar = 100 μm). **(E)** THP-1 was treated with 185 ng/ml PMA for 24 h. And then, coculture it with supernate from U87MG medium for 72 h The flow cytometry showed the expression levels of CD11b and CD206 in these cells. Bar plot showed the proportion of M2 macrophages (CD11b^+^/CD206^+^). **p < 0.01, ***p < 0.001, ****p < 0.0001. **(F)** Immunofluorescence staining showed the expression levels and co-localization of MDK and CD206 in normal brain tissues and GBM tissues.

Research reported that *LRP1* attenuates proinflammatory macrophage activation as receptor for *MDK* (22). And our prementioned cell communication analysis found MK signaling pathway network (*MDK*, *PTPRZ1*, *NCL*, *ITGA6*, *ITGB1*, *LRP1*, and *SDC4*) in GBM samples. We assumed that the MK network (*MDK*/*LRP1*) played a significant role regulating macrophage activation in GBM. First, we collected four samples (Normal = 2; GBM = 2). IHC analysis showed a trend that the expression of *MDK* and *LRP1* was higher in GBM patients (**Figure 5A**). To further validate our hypothesis, we first knockdown the expression of MDK in U87MG cell line with siRNA (**Figure 5B**), and coculture its supernate with induced THP-1. Tanswell assay revealed that compared with negative control (siCon), the knockdown of MDK (siMDK) significantly deceased the migration of macrophages (**Figure 5C**). Then, we induced THP-1 (human monocyte line) into macrophages in the upper chamber of transwell unit, and added recombinant protein MDK into the lower chamber of it. Interestingly, the addition of MDK protein showed significant effect on macrophages migration as well (**Figure 5D**). To assess the effect of MDK on the polarization of macrophages, we cocultured induced THP-1 with supernate from GBM in siMDK and siCon groups respectively. Flow cytometry showed less M2 polarization (CD11b^+^/CD206^+^) in the siMDK group, compared with siCon group (**Figure 5E**). The results suggested that *MDK* significantly induces an immunosuppressive macrophage differentiation. Finally, we conducted immunofluorescence staining to explore the co-localization of MDK and CD206 (marker of M2 macrophages). Results revealed more co-localization of *MDK* and *CD206* in GBM tissues compared with that in normal brain tissues (**Figure 5F**).

### m^6^A Regulators Correlate With ICPs in Immune Microenvironment

Having demonstrated that m^6^A regulators were associated with immune related process, we next addressed whether m^6^A regulators were correlated with ICPs in the GBM microenvironment (**Supplementary Figure 11**). Our results revealed that in GBM cancer cells, expression of *CD274* (PD-L1), *PDCD1LG2* (PD-L2), *LGALS9* (Galectin-9), and *PVR* (CD155) were positively correlated with the expression of multiple m^6^A regulators, especially *YTHDF2*, *YTHDF3*, *LRPPRC*, *METTL3*, *RBM15B*, *FTO*, and *ALKBH5* (**Supplementary Figures 11A, B**). In M2 macrophages, *LGALS9*, *CD86*, and *PVR* showed positive correlations with *YTHDC1*, *YTHDC2*, *YTHDF2*, *YTHDF3*, *ELAVL1*, *LRPPRC*, *METTL3*, *METTL14*, *ZC3H13*, and *ALKBH5* (**Supplementary Figures 11E, F**). Also, we found positive correlations between *HAVCR2* (TIM-3) and *YTHDF2* (**Supplementary Figures 11G, H**) in T/NK cells. These results indicated that m^6^A modification may upregulate these suppressive immune check points and further promote the immunosuppressive microenvironment.

### Bulk RNA-seq Analysis Identifies Two m^6^A-ICP Expression Patterns

**Figure 6:**
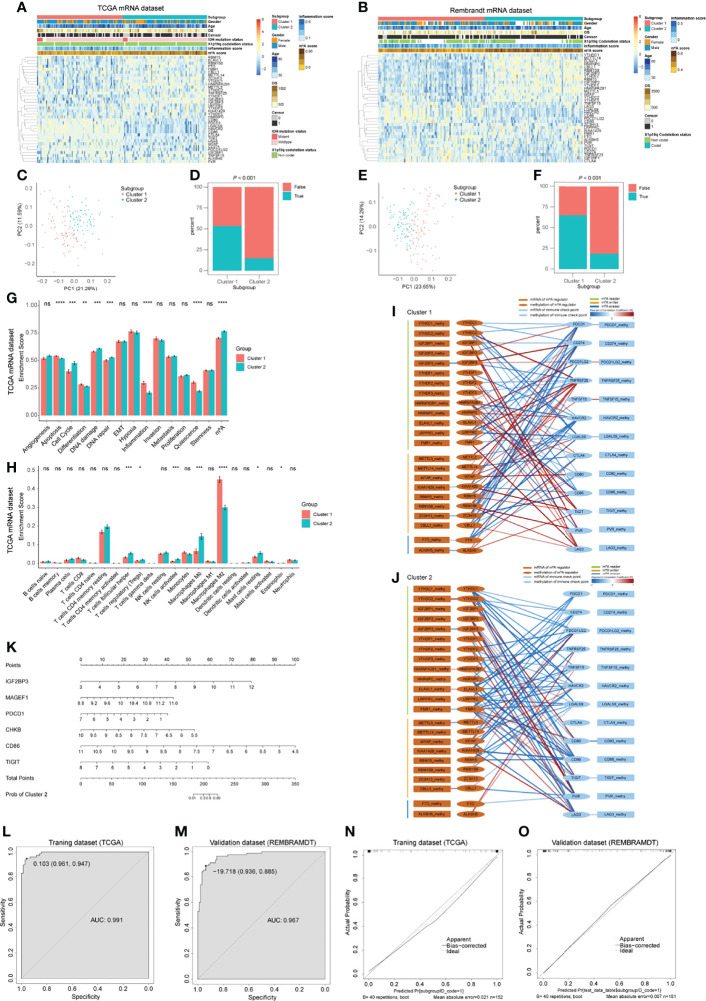
Bulk RNA-seq analysis for GBM patients and predictive model construction and validation. **(A, B)** Heatmap of m^6^A regulators and immune checkpoints (ICPs) revealed the different m^6^A-ICP expression patterns for 2 clusters identified by consensus clustering (for TCGA **(A)** and REMBRANDT **(B)** datasets separately). **(C, E)** Principal component analysis of 2 identified clusters (for TCGA **(C)** and REMBRANDT **(E)** datasets separately). **(D, F)** Predicted potential therapeutic response of ICP inhibitors of 2 identified clusters (for TCGA **(D)** and REMBRANDT **(F)** datasets separately). **(G)** Bar charts illustrating the differences of functional state scores between 2 identified clusters for TCGA dataset. **(H)** Bar charts illustrating the differences of CIBERSORT scores between 2 identified clusters for TCGA dataset. ns: *P* > 0.05, *P ≤ 0.05, **P ≤ 0.01, ***P ≤ 0.001, ****P ≤ 0.0001. **(I, J)** Correlation network of the expression levels and methylation levels of m^6^A regulators and ICPs in 2 identified clusters. **(K)** The nomogram for distinguishing 2 identified clusters. **(L, M)** ROC curves of the nomogram distinguishing 2 identified clusters (L for training dataset and G for validation dataset). **(N, O)** Calibrate plots of the nomogram distinguishing 2 identified clusters (N for training dataset and O for validation dataset).

As we have determined that ICPs in the GBM microenvironment were correlated with m^6^A regulators, we next explored the m^6^A-ICP expression pattern in bulk RNA-seq profiles. According to the similarities of expression of 23 m^6^A regulators and 13 ICPs, we performed consensus clustering, an unsupervised clustering algorithm, in TCGA, CGGA and REMBRANDT datasets, respectively. The optimal clustering stability was obtained when K = 2 (**Supplementary Figures 12A–F**; **Supplementary Figures 15A–C**), and samples were clustered into two subgroups with different distinct features. Cluster 1 showed a low m^6^A/high ICP expression pattern in the heatmap of expression levels. A high m^6^A/low ICP expression pattern was observed for cluster 2 (**Figures 6A, B**; **Supplementary Figure 15D**). PCA for expression levels of m^6^A regulators and ICPs revealed prominent differences between these 2 subgroups (**Figures 6C, E**; **Supplementary Figure 15E**). Notably, the predicted potential therapeutic response of ICP inhibitors, conducted with the TIDE algorithm, was significantly different between these 2 subgroups in TCGA and REMBRANDT datasets (**Figures 6D, F**; *P* < 0.001). Patients in cluster 1 were more likely to response to ICP inhibitor therapy (> 50% were predicted responders), but fewer than 25% patients in cluster 2 were predicted to response to this immunotherapy. In the CGGA datasets, although no statistical significance was observed between these two clusters, cluster 1 showed a trend with higher response proportion to ICP inhibitor therapy (**Supplementary Figure 15F**). However, the overall survival and clinical characteristics, including age, gender, IDH mutation status, and X1p19q codeletion status, were similar between these two clusters (**Supplementary Figures 12G–J**).We further assessed the potential difference of functional states between these 2 clusters. An ssGSEA analysis detected consistent trends between TCGA and REMBRAMDT datasets with statistical significance in the following functional states: apoptosis, cell cycle, DNA damage, DNA repair, inflammation, quiescence and m^6^A modification (**Figure 6G**; **Supplementary Figures 13A**). In order to explore the immune infiltration of these 2 clusters, we utilized the CIBERSORT algorithm and compared the enrichment scores of 22 types of immune cells between these 2 clusters. Interestingly, in both TCGA and REMBRAMDT datasets, the proportion of follicular helper T cells in cluster 1 was lower than that in cluster 2, and there were more M2 macrophages in cluster 1 compared with cluster 2 (**Figure 6H**; **Supplementary Figure 13B**).

In revealing differences between the two clusters at the gene level, we detected 224 up-regulated DEGs and 33 down-regulated DEGs in cluster 1 compared with cluster 2 in TCGA dataset (**Supplementary Figure 13E**). However, no DEGs were found in the REMBRAMDT dataset. Enrichment analyses of these 224 up-regulated DEGs uncovered immune-related BP terms (**Supplementary Figure 13F**). KEGG analysis on the up-regulated DEGs was also conducted (**Supplementary Figure 13G**). We further performed PPI network analyses for all DEGs, and the “cytoHubba” plugin from Cytoscape software was used to reveal hub genes. *CASR*, *CCL19*, *CCR8*, *CCL13*, *RLN3*, *TAS2R42*, *SSTR4*, *MTNR1A*, and *RXFP3* were detected as hub genes (**Supplementary Figure 13H**).

In order to address the potential regulatory mechanism between m^6^A regulators and ICPs at the multiomics level, samples with mRNA-seq profiles and DNA methylation profiles from TCGA were extracted for Pearson correlation analysis. Complex correlation networks of the expression levels and methylation levels of m^6^A regulators and ICPs for all samples and two clusters, respectively, were constructed (**Supplementary Figure 14A**; **Figures 6I, J**). Extensive correlation between expression levels of m^6^A regulators and ICPs were observed, and significant negative correlations between expression levels and DNA methylation levels were detected in *IGF2BP3*, *METTLE3*, *ALKBH5*, *CTLA4*, and *TIGIT* (**Supplementary Figure 14A**). In the subnetworks of these 2 clusters, more positive correlations between expression levels of m^6^A regulators and ICPs were found in cluster 1. For *IGF2BP3*, a negative correlation between expression levels and DNA methylation levels were consistent in these two clusters. However, other statistically significant negative correlations between expression levels and DNA methylation levels were different between cluster 1 and 2 (**Figures 6I, J**). These results suggested different underlying mechanisms of the expression and DNA methylation of m^6^A regulators and ICPs between cluster 1 and 2 GBM patients.

### Construction and Validation of Predictive Model

Having demonstrated that these 2 clusters had distinct features, we next constructed and validated a predictive nomogram model to distinguish these 2 clusters. The LASSO algorithm identified 32 genes, with the optimal λ being 0.02800602 (**Supplementary Figures 14B, C**). Then, a best subset selection analysis was performed to determine the final model. Because of the limitations of the computing power of our device, we only analyzed models with up to 16 variables. The final model contains the following 6 variables: *IGF2BP3*, *MAGEF1*, *PDCD1*, *CHKB*, *CD86*, and *TIGIT*. The final multivariable logistic regression model was visualized as a nomogram (**Figure 6K**). The nomogram showed excellent discrimination performance in TCGA (training cohort, area under curve (AUC) = 0.991), REMBRANDT (validation cohort, AUC = 0.967) and CGGA (validation cohort, AUC = 0.721) datasets (**Figures 6L, M**; **Supplementary Figure 15G**). Calibration plots were drawn, and the calibration was as expected in both training and validation cohorts (**Figure 6N, O**; **Supplementary Figure 15H**). These evaluations proved that the nomogram model performed well in distinguishing these 2 GBM clusters.

## Discussion

Treatment, especially immunotherapy, for GBM remains a challenge (1, 2). The role of m^6^A modification has been shown to be involved in cancer biology (4) and mediating immunotherapy resistance (6, 7). Therefore, there is an urgent need to apply new technologies, including single-cell analysis, to explore potential mechanisms of m^6^A modification in the GBM microenvironment. However, to date, no study has provided an m^6^A modification landscape for GBM microenvironment at the single-cell level. In this study, we retrieved single-cell RNA-seq datasets and used them to identify 7 types of cells in the GBM microenvironment and evaluated the associations between m^6^A regulators and functional states, potential BP, cell communication, and ICPs for these cell types. We discovered that m^6^A modification facilitates the stemness state in GBM cancer cells and promotes an immunosuppressive microenvironment through ICPs and the GALECTIN signaling pathway network. With bulk RNA-seq datasets, we further identified 2 clusters of patients with distinct m^6^A-ICP expression patterns (low m^6^A/high ICP and high m^6^A/low ICP) and predicted the responses to ICP inhibitors. A well performing nomogram model was constructed to distinguish these 2 GBM clusters.

The high inter-tumor heterogeneity of GBM immune microenvironment has reached a broad consensus. In the current research, we observed high inter-tumor heterogeneity of M1 and M2 macrophage proportions (**Figure 2A**). In around half of included samples, the infiltration proportions of M2 macrophage were higher than that of M1 macrophages. And higher proportions of M1 macrophages were found in the other samples (**Figure 2I**). Sørensen et al. (23) reported interesting phenomenon that on average 44% and 8% of the tumor associated macrophages (TAM) expressed the marker HLA-DR and TNF-α for M1 macrophages, respectively, while 10% and 3% of TAMs expressed IL10 and TGF-β1, which were markers for M2 macrophages. Also, these proportions varied greatly among different samples. These results indicated that the proportions of M1 and M2 macrophages in GBM have high inter-tumor heterogeneity.

There has been limited discussion about the distribution of expression of m^6^A regulators in different cell types and throughout cell cycle phases. The bulk RNA-seq dataset revealed that most m^6^A regulators have relatively high expression levels in GBM compared with levels in normal samples. This finding was especially apparent for *HNRNPA2B1*, *HNRNPC* and *WTAP* (**Supplementary Figure 2J**). A previous study has shown increased expression of *METTL3* and decreased expression levels of *METTL14* and *ALKBH5* and no significantly changes to the expression of *FTO* in GSCs (8). Another research detected elevated *ALKBH5* in GSCs (24). The present study detected the expression levels of 23 m^6^A regulators in 7 types of cells and 3 cell cycle phases. We found that expression levels of m^6^A regulators were higher in M1 macrophages, GBM cancer cells, and T/NK cells than in other types of cells (**Figure 3A**), and cells at G_1_ phase showed higher expression levels of m^6^A regulators relative to that of cells at S and G_2_M phases (**Figure 3R**). *HNRNPA2B1* and *HNRNPC* had obviously higher expression levels than other m^6^A regulators in all 7 types of cells and 3 cell cycle phases. Thus, besides the intratumor heterogeneity of m^6^A regulators, we also detected obvious intertumor heterogeneity of cell types and cell cycle phases in different samples (**Figure 3Q**; **Supplementary Figure 3H**). These results suggested that the roles of m^6^A modification in GBM may be different in different types of cells, and further investigations of m^6^A modifications in GBM should take cell type into consideration.

Multiple studies have analyzed the mechanisms by which m^6^A modification maintains stemness of GSCs. For example, *ALKBH5* promotes stemness of GSCs by sustaining *FOXM1* expression (24, 25) and *METTL3 (* 19), *HNRNPA2B1* and *HNRNPC (* 26) maintain stemness of GSCs by targeting *SOX2*. The product of the *SOX2* gene is an oncogenic transcription factor in many cancers; accordingly, in the current research, we found significant positive correlations between *SOX2* and *YTHDC1*, *IGF2BP2*, *HNRNPC*, *ELAVL1*, *FMR1*, *KIAA1429*, and *ZC3H13* in GBM cancer cells (**Figure 4D, E**). Among these m^6^A regulators, IGF2BP2 had been proven to be a downstream m^6^A reader of *SOX2* in colorectal carcinoma (20). Our results suggested that *YTHDC1*, *IGF2BP2*, *HNRNPC*, *ELAVL1*, *FMR1*, *KIAA1429*, and *ZC3H13* may have potential interactions with *SOX2* and further promote stemness of GBM cancer cells. The research of Su et al. demonstrated that *FTO* plays a carcinogenic role maintaining the self-renewal ability of GSCs *via FTO*/m^6^A/*MYC*/*CEBPA* signaling (27). Importantly, *MYC* is a commonly activated oncogene in human cancer. Our correlation analysis uncovered positive correlations between *MYC* and *IGF2BP1*, *YTHDF1*, *YTHDF2*, *YTHDF3*, *HNRNPC*, *LRPPRC*, *METTL3*, *WTAP*, *KIAA1429*, *RBM15*, *RBM15B*, *CBLL1*, *FTO*, and *ALKBH5* (**Figure 4D, E**). The *IGF2BP1* protein has been proposed to protect *MYC* mRNA from endonucleolytic attack (28). These results indicated a more extensive potential stemness regulation network between MYC and these m^6^A regulators in GBM cancer cells.

The m^6^A modification was reported to play a role in programmed cell death, including apoptosis (9, 29–32), ferroptosis (33, 34) and pyroptosis (35, 36). These studies mainly focused on cancer cells, and none of them have discussed the immune cells in the tumor microenvironment. In our research, we found that the m^6^A score was significantly correlated with apoptosis, ferroptosis and pyroptosis in M1 macrophages and apoptosis and pyroptosis in T/NK cells in GBM microenvironment (**Figures 4A, B**). Further correlation analysis of m^6^A regulators and these programmed cell death-related gene sets were visualized as regulation networks (**Supplementary Figures 4B–D, H, J**). These results indicated a potentially complex mechanism of m^6^A modification-mediated programmed cell death in M1 macrophages and T/NK cells. It remains unknown whether programmed cell death in these immune cells could lead to the immunosuppressive microenvironment in GBM or not. And the regulatory role of m^6^A regulators in these processes is still unclear. Further research is needed in order to verify these findings and to analyze its impact on the GBM microenvironment.

The GALECTIN signaling pathway network includes 3 ligand-receptor pairs (*LGALS9*-*CD44*, *LGALS9*-*CD45*, *LGALS9*-*HAVCR2*; **Supplementary Table S6**). *LGALS9* (Galectin-9) and *HAVCR2* (TIM-3) are known as immune check points. *CD45* (PTPRC) was an essential regulator of T cell activation (37), and *CD44* is involved in diverse cellular processes including proliferation, apoptosis, and angiogenesis (38). Limited studies have discussed the role of m^6^A modification in the GALECTIN network. Wang et al. reported that *IGF2BP1* stabilizes mRNA transcripts of *CD44* and further promotes proliferation and invasion of GBM (39). A study from Lin et al. found positive correlations between expression of *YTHDF2* and *HAVCR2* in lower-grade glioma (40). In the current research, we observed robust occurrence of the GALECTIN network in included samples (**Supplementary Figures 6A, B**), which indicated that the GALECTIN network plays an important role in the GBM microenvironment. The network mainly started from M1 and M2 macrophages and was targeted at other types of cells in the GBM microenvironment. Relatively high expression levels of genes in the GALECTIN network were detected in M1 macrophages, M2 macrophages, and T/NK cells (**Supplementary Figure 10C**). Further correlation analysis found extensive significant correlations between m^6^A regulators and genes in the GALECTIN network (**Supplementary Figures 7C, D**; **Supplementary Figure 8**). These results indicated that the m^6^A modification was involved in the potential regulatory mechanism of the GALECTIN network. However, the detailed regulatory pathways between m^6^A modification and the GALECTIN network is still known. And whether inhibiting the GALECTIN network by regulation of m^6^A modification could lead to better prognosis of GBM patients remains to be further verified.

We conducted a series of *in vitro* experiments to validate the regulation role of *MDK* in GBM microenvironment (**Figure 5**). Based on our results, *MDK*, which was secreted by GBM cancer cells, induced the migration and immunosuppressive polarization of macrophages. Our results were consistent with the role of MDK identified by Zhang et al. in 2021 (41). In their gallbladder cancer research, *MDK* interacts with its receptor *LRP1*, which is expressed by tumor-infiltrating macrophages, and further promotes immunosuppressive macrophage differentiation. We assume that MDK could be a treatment target for GBM, further *in vivo* validation and translational research are expected to develop a novel therapy for GBM.

Rapid progress in cancer immunotherapy, especially in the development of ICP inhibitors, has revolutionized the treatment of many solid tumors and driven the study of immunotherapy in glioma. However, the effect of ICP blockades in GBM has not been satisfactory (42). Some studies have found that m^6^A modification was correlated with immune infiltration in glioma (43–47). However, to date, no research has analyzed the relationship between m^6^A modification and ICPs in GBM. In the current study, we revealed extensive correlations between m^6^A regulators and ICPs in GBM cancer cells, M2 macrophages, and T/NK cells (**Supplementary Figure 11**). Notably, in GBM cancer cells, *CD274* (PD-L1), *PDCD1LG2* (PD-L2), *LGALS9* (Galectin-9), and *PVR* (CD155) were correlated with m^6^A regulators. In the bulk RNA-seq analysis, we identified 2 clusters of patients. Cluster 1 patients (low m^6^A/high ICP) showed higher predicted response rates to ICP inhibitors, and cluster 2 patients (high m^6^A/low ICP) had lower predicted response rates (**Figures 6A, B, D, F**). Although these 2 clusters showed different m^6^A-ICP expression patterns, they had similar overall survival times (**Supplementary Figures 12I, J**). These results indicated that these 2 clusters may have different mechanisms for immune escape and require different treatment strategies.

In the current research, the LASSO analysis and best subset selection analysis filtered 6 genes, which could distinguish these 2 clusters. Among them, IGF2BP3 was a m^6^A reader. PDCD1, CD86, and TIGIT were significant ICPs. MAGEF1 (melanoma antigen family F1) is a member of MAGE family and belongs to type 2 MAGE (T2M) category (48). Arora et al. ’s research found that in glioma downregulation of T2Ms was associated with immune infiltration and poor overall survival (48). Weon et al. reported that MAGEF1 alters DNA repair enzymes *via* the cytosolic iron-sulfur cluster assembly (CIA) pathway (49). And MAGEF1 was highly amplified in multiple human cancer types and related with increased mutational burden (49). Based on these findings, MAGEF1 might related with ICPs *via* m6A modification or CIA pathways, which needed to be further explored. CHKB (choline kinase beta) played a key role maintaining the normal phosphatidylcholine level (50). The relationship between CHKB and glioma haven’t been explored. Our results showed that lower expression level of CHKB suggested higher possibility of cluster 2 (high m^6^A/low ICP; **Figure 6K**). However, the potential mechanisms need to be further analyzed.

Based on the correlations between m^6^A regulators and ICPs found in the current study, we suggest that by targeting these related m^6^A regulators, researchers may downregulate the expression of these immune suppressive ICPs and further enhance the efficacies of ICP inhibitors for cluster 2 patients. Further research is needed to verify these findings and to specify how it influences strategies directing the use of ICP inhibitors for GBM.

Research have found that tumors with extensive infiltration of immunosuppressive macrophages are refractory to ICP inhibitor therapy (51, 52). However, contrary results were observed in our research. TIDE algorithm predicted that cluster 1 was more responsive to ICP inhibitors (**Figures 6D, F**), and CIBERSORT algorithm showed higher M2 macrophage infiltration in cluster 1(**Figure 6H**; **Supplementary Figure 13B**). These results may reflect the inner complexity of GBM. Further high-quality research of ICP inhibitors in GBM are expected.

The current research revealed the m^6^A regulators expression landscape of GBM at single-cell level. Our research suggested that by altering the m^6^A modification in GBM, researchers may be able to influence the stemness status and immunosuppressive microenvironment of GBM. Combined with immunotherapy, these regulations have potential to advance the treatment effect of immunotherapy.

Some limitations in the current study should be acknowledged. First, as a retrospective study, these analyses were conducted using published datasets, and the results about potential mechanisms of action of m^6^A modifications in the GBM microenvironment need further experimental validation. Second, we were not able to identify clusters of GSCs and to separate the T/NK cluster into more detailed clusters. Here, the problem may be due to limited cell numbers for these cell types. Third, missing data was observed after merging the 5 single-cell datasets. However, the data of m^6^A regulators was almost complete, and only a few datasets partially lacked genes for some ICPs (**Supplementary Table S3**). Also, the information regarding the rest of the genes is complete. To avoid the influence of the missing data in the analysis, we excluded samples with missing data in the analyses of correlations between m^6^A regulators and ICPs (**Supplementary Figure 11**).

## Conclusion

Through analyses at the single-cell level, for the first time, we discovered that m^6^A modification facilitates stemness state in GBM cancer cells and promotes the immunosuppressive microenvironment through ICPs and the GALECTIN signaling pathway network in the GBM microenvironment. We further identified 2 clusters of patients with distinct m^6^A-ICP expression patterns (low m^6^A/high ICP and high m^6^A/low ICP) and predicted the response of ICP inhibitors in bulk RNA-seq analysis. A well performing nomogram model was constructed to distinguish these 2 GBM clusters. We hope the novel understanding of the roles of m^6^A modification in the GBM microenvironment may assist the development of immunotherapy and precision treatment in GBM.

## Data Availability Statement

Publicly available datasets were analyzed in this study. This data can be found here: The data analyzed in this study were obtained from Gene Expression Omnibus (GEO) at GSE141383, GSE138794, GSE84465, GSE103224, GSE89567, GSE124731 and GSE108474 (REMBRANDT), and from the Cancer Genome Atlas (TCGA, https://portal.gdc.cancer.gov/) and Chinese Glioma Genome Atlas (CGGA, http://www.cgga.org.cn/).

## Ethics Statement

The studies involving human participants were reviewed and approved by Jinling Hospital research ethics board. The patients/participants provided their written informed consent to participate in this study.

## Author Contributions

CM, XC and SZ were involved in conception and design of the study. FY, XC, YG, YA, CD, JZ, JY, CT, and ZC were involved in development of methodology, acquisition of data, analysis and interpretation of data. XC was involved in writing of the manuscript. YW and AZ contributed to the interpretation of the experimental results. CM oversaw the conception and design of the study, interpretation of data and writing of the manuscript. All authors contributed to the article and approved the submitted version.

## Conflict of Interest

The authors declare that the research was conducted in the absence of any commercial or financial relationships that could be construed as a potential conflict of interest.

## Publisher’s Note

All claims expressed in this article are solely those of the authors and do not necessarily represent those of their affiliated organizations, or those of the publisher, the editors and the reviewers. Any product that may be evaluated in this article, or claim that may be made by its manufacturer, is not guaranteed or endorsed by the publisher.

## Supplementary Material

Supplementary Figure 1 Quality control of samples from **(A)** GSE141383, **(B)** GSE138794, **(C)** GSE84465, **(D)** GSE103224, and **(E)** GSE89567 datasets.

Supplementary Figure 2 Distribution of m^6^A regulators in GBM microenvironment. **(A–I)** tSNE plots of 9 m^6^A regulators. **(J)** Expression levels of 23 m^6^A regulators in GBM and normal samples from bulk RNA-seq dataset in GEPIA website.

Supplementary Figure 3 Expression levels of m^6^A regulators in 3 cell cycles. **(A-G)** Expression levels of m^6^A regulators in 3 stages of the cell cycle from GBM cancer cells **(A)**, oligodendrocytes **(B)**, astrocytes **(C)**, M1 macrophages **(D)**, M2 macrophages **(E)**, T/NK cells **(F)**, and endothelial cells **(G)**. **(H)** Distribution of 3 stages of the cell cycle in each included sample.

Supplementary Figure 4 correlation network of m^6^A regulators and functional states. **(A)** correlation network of m^6^A regulators and differentiation related genes in GBM cancer cells. **(B-D)** correlation network of m^6^A regulators and apoptosis **(B)**, ferroptosis **(C)**, and pyroptosis **(D)** related genes in M1 macrophages. **(E-G)** correlation network of m^6^A regulators and EMT **(E)**, invasion **(F)**, and metastasis **(G)** related genes in M2 macrophages. **(H–J)** correlation network of m^6^A regulators and apoptosis **(H)**, DNA damage **(I)**, and pyroptosis **(J)** related genes in T/NK cells. **(K-M)** correlation network of m^6^A regulators and angiogenesis **(K)**, invasion **(L)**, and metastasis **(M)** related genes in endothelial cells.

Supplementary Figure 5 m^6^A related gene clusters and functional annotation. **(A-C)** PPI networks of cluster 1 **(A)**, cluster 2 **(B)**, and cluster 3 **(C)** of m^6^A related genes in GBM cancer cells. **(D–F)** PPI networks of cluster 1 **(D)**, cluster 2 **(E)**, and cluster 3 **(F)** of m^6^A related genes in M1 macrophages. **(G-I)** PPI networks of cluster 1 **(G)**, cluster 2 **(H)**, and cluster 3 **(I)** of m^6^A related genes in M2 macrophages. **(J, K)** PPI networks of cluster 1 **(J)**, and cluster 2 **(K)** of m^6^A related genes in T/NK cells. **(L-N)** PPI networks of cluster 1 **(L)**, cluster 2 **(M)**, and cluster 3 **(N)** of m^6^A related genes in endothelial cells. **(O-Q)** PPI network and enriched biological process terms of cluster 1 **(O)**, cluster 2 **(P)**, and cluster 3 **(Q)** of m^6^A related genes in oligodendrocytes.

Supplementary Figure 6 Cell communication analysis reveals robust signal pathways in the GBM cancer microenvironment. **(A, B)** The GALECTIN signaling pathway network as detected in cell communication analyses for all samples **(A)** and each sample **(B)**. **(C, D)** The GRN signaling pathway network as detected in cell communication analyses for all samples **(C)** and each sample **(D)**. **(E, F)** The PTN signaling pathway network as detected in cell communication analyses for all samples **(E)** and each sample **(F)**. **(G, H)** The SPP1 signaling pathway network as detected in cell communication analyses for all samples **(G)** and each sample **(H)**. **(I, J)** The ANNEXIN signaling pathway network as detected in cell communication analyses for all samples **(I)** and each sample **(J)**. **(K, L)** The VISFATIN signaling pathway network as detected in cell communication analyses for all samples **(K)** and each sample **(L)**. **(M, N)** The MK signaling pathway network as detected in cell communication analyses for all samples **(M)** and each sample **(N)**.

Supplementary Figure 7 Correlation analysis of m^6^A regulators and genes in signaling pathway networks in GBM cancer cells and M1 macrophages. **(A, B)** Correlation heatmap **(A)** and correlation analysis **(B)** of m^6^A regulators and genes in signaling pathway networks in GBM cancer cells. **(C, D)** Correlation heatmap **(C)** and correlation analysis **(D)** of m^6^A regulators and genes in signaling pathway networks in M1 macrophages. NS: *P* > 0.05, **P* ≤ 0.05, ***P* ≤ 0.01, ****P* ≤ 0.001.

Supplementary Figure 8 Correlation analysis of m^6^A regulators and genes in signaling pathway networks in M2 macrophages and T/NK cells. **(A, B)** Correlation heatmap **(A)** and correlation analysis **(B)** of m^6^A regulators and genes in signaling pathway networks in M2 macrophages. **(C, D)** Correlation heatmap **(C)** and correlation analysis **(D)** of m^6^A regulators and genes in signaling pathway networks in T/NK cells. NS: *P* > 0.05, **P* ≤ 0.05, ***P* ≤ 0.01, ****P* ≤ 0.001.

Supplementary Figure 9 Correlation analysis of m^6^A regulators and genes in signaling pathway networks in endothelial cells and astrocytes. **(A, B)** Correlation heatmap **(A)** and correlation analysis **(B)** of m^6^A regulators and genes in signaling pathway networks in endothelial cells. **(C, D)** Correlation heatmap **(C)** and correlation analysis **(D)** of m^6^A regulators and genes in signaling pathway networks in astrocytes. NS: *P* > 0.05, **P* ≤ 0.05, ***P* ≤ 0.01, ****P* ≤ 0.001.

Supplementary Figure 10 Correlation analysis of m^6^A regulators and genes in signaling pathway networks in oligodendrocytes. **(A, B)** Correlation heatmap **(A)** and correlation analysis **(B)** of m^6^A regulators and genes in signaling pathway networks in oligodendrocytes. **(C)** Expression levels of genes in signaling pathway networks in 7 cell types. NS: *P* > 0.05, **P* ≤ 0.05, ***P* ≤ 0.01, ****P* ≤ 0.001.

Supplementary Figure 11 Correlation analysis of m^6^A regulators and immune checkpoints (ICPs). **(A, B)** Correlation heatmap **(A)** and correlation analysis **(B)** of m^6^A regulators and ICPs in GBM cancer cells. **(C, D)** Correlation heatmap **(C)** and correlation analysis **(D)** of m^6^A regulators and ICPs in M1 macrophages. **(E, F)** Correlation heatmap **(E)** and correlation analysis **(F)** of m^6^A regulators and ICPs in M2 macrophages. **(G, H)** Correlation heatmap **(G)** and correlation analysis (H) of m^6^A regulators and ICPs in T/NK cells. **(I, J)** Correlation heatmap **(I)** and correlation analysis (J) of m^6^A regulators and ICPs in endothelial cells. **(K, L)** Correlation heatmap (K) and correlation analysis (L) of m^6^A regulators and ICPs in astrocytes. **(M, N)** Correlation heatmap (M) and correlation analysis (N) of m^6^A regulators and ICPs in oligodendrocytes. NS: *P* > 0.05, **P* ≤ 0.05, ***P* ≤ 0.01, ****P* ≤ 0.001.

Supplementary Figure 12 Identification and validation of 2 clusters of GBM patients in bulk RNA-seq analysis. **(A, D)** Consensus clustering matrix for k = 2, which was the optimal cluster number in the TCGA dataset **(A)** and REMBRANDT dataset **(D)**. **(B, E)** CDF curves of the consensus score (k = 2-6) in the TCGA dataset **(B)** and REMBRANDT dataset **(E)**. **(C, F)** Relative change in the area under the CDF curve (k = 2-6) in the TCGA dataset **(C)** and REMBRANDT dataset **(F)**. **(G, H)** Clinical features of 2 identified clusters in the TCGA dataset **(G)** and REMBRANDT dataset **(H)**. **(I, J)** Kaplan-Meier survival analyses of 2 identified clusters in the TCGA dataset **(I)** and REMBRANDT dataset **(J)**

Supplementary Figure 13 comparison of 2 identified clusters of GBM patients in bulk RNA-seq analysis. **(A)** Bar charts illustrating the differences of functional state scores between 2 identified clusters for REMBRANDT dataset. **(B)** Bar charts illustrating the differences of functional state scores between 2 identified clusters for REMBRANDT dataset. **(C, D)** Bar charts illustrating the differences of expression levels of m^6^A regulators, immune checkpoints, and genes in detected cell communication signaling pathway networks between 2 identified clusters for TCGA **(C)** and REMBRANDT **(D)** dataset. **(E)** Volcano plot of differentially expressed genes (DEGs) between 2 identified clusters in TCGA dataset. **(F, G)** GO **(F)** and KEGG **(G)** analyses of up-regulated DEGs in TCGA dataset. **(H)** PPI network showing hub genes of up-regulated DEGs in TCGA dataset. ns: *P* > 0.05, **P* ≤ 0.05, ***P* ≤ 0.01, ****P* ≤ 0.001, *****P* ≤ 0.0001.

Supplementary Figure 14 Multiomic analysis and LASSO analysis. **(A)** Correlation network of the expression levels and methylation levels of m^6^A regulators and ICPs in all GBM samples from TCGA dataset. **(B, C)** LASSO regression analysis of training dataset (TCGA).

Supplementary Figure 15 Bulk RNA-seq analysis with CGGA dataset. **(A),** Consensus clustering matrix for k = 2, which was the optimal cluster number in the CGGA dataset. **(B)** CDF curves of the consensus score (k = 2-6) in the CGGA dataset. **(C)** Relative change in the area under the CDF curve (k = 2-6) in the CGGA dataset. **(D)** Heatmap of m^6^A regulators and immune checkpoints (ICPs) revealed the different m^6^A-ICP expression patterns for 2 clusters identified by consensus clustering for CGGA dataset. **(E)** Principal component analysis of 2 identified clusters for CGGA dataset. **(F)** Predicted potential therapeutic response of ICP inhibitors of 2 identified clusters for CGGA dataset. **(G)** ROC curve of the nomogram distinguishing 2 identified clusters for validation dataset (CGGA). **(H)** Calibrate plot of the nomogram distinguishing 2 identified clusters for validation dataset (CGGA).

## References

[1] Wu W Klockow JL Zhang M Lafortune F Chang E Jin L. Glioblastoma Multiforme (GBM): An Overview of Current Therapies and Mechanisms of Resistance. Pharmacol Res (2021) 171:105780. doi: 10.1016/j.phrs.2021.105780 34302977 PMC8384724

[2] Ostrom QT Cioffi G Gittleman H Patil N Waite K Kruchko C. CBTRUS Statistical Report: Primary Brain and Other Central Nervous System Tumors Diagnosed in the United States in 2012-2016. Neuro Oncol (2019) 2:1: v1–v100. doi: 10.1093/neuonc/noz150 PMC6823730 31675094

[3] Ott M Prins RM Heimberger AB. The Immune Landscape of Common CNS Malignancies: Implications for Immunotherapy. Nat Rev Clin Oncol (2021) 18:794–44. doi: 10.1038/s41571-021-00518-9 PMC11090136 34117475

[4] Zaccara S Ries RJ Jaffrey SR. Reading, Writing and Erasing mRNA Methylation. Nat Rev Mol Cell Biol (2019) 20:608–24. doi: 10.1038/s41580-019-0168-5 31520073

[5] Huang W Chen TQ Fang K Zeng ZC Ye H Chen YQ. N6-Methyladenosine Methyltransferases: Functions, Regulation, and Clinical Potential. J Hematol Oncol (2021) 14:117. doi: 10.1186/s13045-021-01129-8 34315512 PMC8313886

[6] Yin H Zhang X Yang P Zhang X Peng Y Li D. RNA M6a Methylation Orchestrates Cancer Growth and Metastasis *via* Macrophage Reprogramming. Nat Commun (2021) 12:1394. doi: 10.1038/s41467-021-21514-8 33654093 PMC7925544

[7] Wang L Hui H Agrawal K Kang Y Li N Tang R. M(6) A RNA Methyltransferases METTL3/14 Regulate Immune Responses to Anti-PD-1 Therapy. EMBO J (2020) 39:e104514. doi: 10.15252/embj.2020104514 32964498 PMC7560214

[8] Zhang Y Geng X Li Q Xu J Tan Y Xiao M. M6a Modification in RNA: Biogenesis, Functions and Roles in Gliomas. J Exp Clin Cancer Res (2020) 39:192. doi: 10.1186/s13046-020-01706-8 32943100 PMC7500025

[9] Li F Zhang C Zhang G. M6a RNA Methylation Controls Proliferation of Human Glioma Cells by Influencing Cell Apoptosis. Cytogenet Genome Res (2019) 159:119–25. doi: 10.1159/000499062 31639789

[10] Chai RC Wu F Wang QX Zhang S Zhang KN Liu YQ. M(6)A RNA Methylation Regulators Contribute to Malignant Progression and Have Clinical Prognostic Impact in Gliomas. Aging (Albany NY) (2019) 11:1204–25. doi: 10.18632/aging.101829 PMC6402513 30810537

[11] Yuan J Levitin HM Frattini V Bush EC Boyett DM Samanamud J. Single-Cell Transcriptome Analysis of Lineage Diversity in High-Grade Glioma. Genome Med (2018) 10:57. doi: 10.1186/s13073-018-0567-9 30041684 PMC6058390

[12] Darmanis S Sloan SA Croote D Mignardi M Chernikova S Samghababi P. Single-Cell RNA-Seq Analysis of Infiltrating Neoplastic Cells at the Migrating Front of Human Glioblastoma. Cell Rep (2017) 21:1399–410. doi: 10.1016/j.celrep.2017.10.030 PMC5810554 29091775

[13] Chen AX Gartrell RD Zhao J Upadhyayula PS Zhao W Yuan J. Single-Cell Characterization of Macrophages in Glioblastoma Reveals MARCO as a Mesenchymal Pro-Tumor Marker. Genome Med (2021) 13:88. doi: 10.1186/s13073-021-00906-x 34011400 PMC8136167

[14] Wang L Babikir H Müller S Yagnik G Shamardani K Catalan F. The Phenotypes of Proliferating Glioblastoma Cells Reside on a Single Axis of Variation. Cancer Discov (2019) 9:1708–19. doi: 10.1158/2159-8290.Cd-19-0329 PMC7161589 31554641

[15] Venteicher AS Tirosh I Hebert C Yizhak K Neftel C Filbin MG. Decoupling Genetics, Lineages, and Microenvironment in IDH-Mutant Gliomas by Single-Cell RNA-Seq. Science (2017) 355:eaai8478. doi: 10.1126/science.aai8478 28360267 PMC5519096

[16] Gusev Y Bhuvaneshwar K Song L Zenklusen JC Fine H Madhavan S. The REMBRANDT Study, a Large Collection of Genomic Data From Brain Cancer Patients. Sci Data (2018) 5:180158. doi: 10.1038/sdata.2018.158 30106394 PMC6091243

[17] Hao Y Hao S Andersen-Nissen E Mauck WM 3rd Zheng S Butler A. Integrated Analysis of Multimodal Single-Cell Data. Cell (2021) 184:3573–87.e29. doi: 10.1016/j.cell.2021.04.048 34062119 PMC8238499

[18] Zhang X Lan Y Xu J Quan F Zhao E Deng C. CellMarker: A Manually Curated Resource of Cell Markers in Human and Mouse. Nucleic Acids Res (2019) 47:D721–d8. doi: 10.1093/nar/gky900 PMC6323899 30289549

[19] Visvanathan A Patil V Arora A Hegde AS Arivazhagan A Santosh V. Essential Role of METTL3-Mediated M(6)A Modification in Glioma Stem-Like Cells Maintenance and Radioresistance. Oncogene (2018) 37:522–33. doi: 10.1038/onc.2017.351 28991227

[20] Li T Hu PS Zuo Z Lin JF Li X Wu QN. METTL3 Facilitates Tumor Progression *via* an M(6)A-IGF2BP2-Dependent Mechanism in Colorectal Carcinoma. Mol Cancer (2019) 18:112. doi: 10.1186/s12943-019-1038-7 31230592 PMC6589893

[21] Gieseke F Kruchen A Tzaribachev N Bentzien F Dominici M Müller I. Proinflammatory Stimuli Induce Galectin-9 in Human Mesenchymal Stromal Cells to Suppress T-Cell Proliferation. Eur J Immunol (2013) 43:2741–9. doi: 10.1002/eji.201343335 23817958

[22] Mantuano E Brifault C Lam MS Azmoon P Gilder AS Gonias SL. LDL Receptor-Related Protein-1 Regulates Nfκb and microRNA-155 in Macrophages to Control the Inflammatory Response. Proc Natl Acad Sci USA (2016) 113:1369–74. doi: 10.1073/pnas.1515480113 PMC4747752 26787872

[23] Sørensen MD Dahlrot RH Boldt HB Hansen S Kristensen BW. Tumour-Associated Microglia/Macrophages Predict Poor Prognosis in High-Grade Gliomas and Correlate With an Aggressive Tumour Subtype. Neuropathol Appl Neurobiol (2018) 44:185–206. doi: 10.1111/nan.12428 28767130

[24] Cui Q Shi H Ye P Li L Qu Q Sun G. M(6)A RNA Methylation Regulates the Self-Renewal and Tumorigenesis of Glioblastoma Stem Cells. Cell Rep (2017) 18:2622–34. doi: 10.1016/j.celrep.2017.02.059 PMC5479356 28297667

[25] Zhang S Zhao BS Zhou A Lin K Zheng S Lu Z. M(6)A Demethylase ALKBH5 Maintains Tumorigenicity of Glioblastoma Stem-Like Cells by Sustaining FOXM1 Expression and Cell Proliferation Program. Cancer Cell (2017) 31:591–606.e6. doi: 10.1016/j.ccell.2017.02.013 28344040 PMC5427719

[26] Fang X Yoon JG Li L Tsai YS Zheng S Hood L. Landscape of the SOX2 Protein-Protein Interactome. Proteomics (2011) 11:921–34. doi: 10.1002/pmic.201000419 21280222

[27] Su R Dong L Li C Nachtergaele S Wunderlich M Qing Y. R-2hg Exhibits Anti-Tumor Activity by Targeting FTO/m(6)A/MYC/CEBPA Signaling. Cell (2018) 172:90–105.e23. doi: 10.1016/j.cell.2017.11.031 29249359 PMC5766423

[28] Bell JL Wächter K Mühleck B Pazaitis N Köhn M Lederer M. Insulin-Like Growth Factor 2 mRNA-Binding Proteins (IGF2BPs): Post-Transcriptional Drivers of Cancer Progression? Cell Mol Life Sci (2013) 70:2657–75. doi: 10.1007/s00018-012-1186-z PMC3708292 23069990

[29] Xi Z Wang P Xue Y Shang C Liu X Ma J. Overexpression of miR-29a Reduces the Oncogenic Properties of Glioblastoma Stem Cells by Downregulating Quaking Gene Isoform 6. Oncotarget (2017) 8:24949–63. doi: 10.18632/oncotarget.15327 PMC5421901 28212562

[30] Vu LP Pickering BF Cheng Y Zaccara S Nguyen D Minuesa G. The N(6)-Methyladenosine (M(6)A)-Forming Enzyme METTL3 Controls Myeloid Differentiation of Normal Hematopoietic and Leukemia Cells. Nat Med (2017) 23:1369–76. doi: 10.1038/nm.4416 PMC5677536 28920958

[31] Park YM Hwang SJ Masuda K Choi KM Jeong MR Nam DH. Heterogeneous Nuclear Ribonucleoprotein C1/C2 Controls the Metastatic Potential of Glioblastoma by Regulating PDCD4. Mol Cell Biol (2012) 32:4237–44. doi: 10.1128/mcb.00443-12 PMC3457347 22907752

[32] Deng J Chen S Wang F Zhao H Xie Z Xu Z. Effects of hnRNP A2/B1 Knockdown on Inhibition of Glioblastoma Cell Invasion, Growth and Survival. Mol Neurobiol (2016) 53:1132–44. doi: 10.1007/s12035-014-9080-3 25586062

[33] Ma L Zhang X Yu K Xu X Chen T Shi Y. Targeting SLC3A2 Subunit of System X(C)(-) is Essential for M(6)A Reader YTHDC2 to be an Endogenous Ferroptosis Inducer in Lung Adenocarcinoma. Free Radic Biol Med (2021) 168:25–43. doi: 10.1016/j.freeradbiomed.2021.03.023 33785413

[34] Song Z Jia G Ma P Cang S. Exosomal miR-4443 Promotes Cisplatin Resistance in Non-Small Cell Lung Carcinoma by Regulating FSP1 M6a Modification-Mediated Ferroptosis. Life Sci (2021) 276:119399. doi: 10.1016/j.lfs.2021.119399 33781830

[35] Guo M Yan R Ji Q Yao H Sun M Duan L. IFN Regulatory Factor-1 Induced Macrophage Pyroptosis by Modulating M6a Modification of Circ_0029589 in Patients With Acute Coronary Syndrome. Int Immunopharmacol (2020) 86:106800. doi: 10.1016/j.intimp.2020.106800 32674051

[36] Diao MY Zhu Y Yang J Xi SS Wen X Gu Q. Hypothermia Protects Neurons Against Ischemia/Reperfusion-Induced Pyroptosis *via* M6a-Mediated Activation of PTEN and the PI3K/Akt/GSK-3β Signaling Pathway. Brain Res Bull (2020) 159:25–31. doi: 10.1016/j.brainresbull.2020.03.011 32200003

[37] Al Barashdi MA Ali A McMullin MF Mills K. Protein Tyrosine Phosphatase Receptor Type C (PTPRC or CD45). J Clin Pathol (2021) 74:548–52. doi: 10.1136/jclinpath-2020-206927 PMC8380896 34039664

[38] Mooney KL Choy W Sidhu S Pelargos P Bui TT Voth B. The Role of CD44 in Glioblastoma Multiforme. J Clin Neurosci (2016) 34:1–5. doi: 10.1016/j.jocn.2016.05.012 27578526

[39] Wang RJ Li JW Bao BH Wu HC Du ZH Su JL. MicroRNA-873 (miRNA-873) Inhibits Glioblastoma Tumorigenesis and Metastasis by Suppressing the Expression of IGF2BP1. J Biol Chem (2015) 290:8938–48. doi: 10.1074/jbc.M114.624700 PMC4423684 25670861

[40] Lin X Wang Z Yang G Wen G Zhang H. YTHDF2 Correlates With Tumor Immune Infiltrates in Lower-Grade Glioma. Aging (Albany NY) (2020) 12:18476–500. doi: 10.18632/aging.103812 PMC7585119 32986017

[41] Zhang Y Zuo C Liu L Hu Y Yang B Qiu S. Single-Cell RNA-Sequencing Atlas Reveals an MDK-Dependent Immunosuppressive Environment in ErbB Pathway-Mutated Gallbladder Cancer. J Hepatol (2021) 75:1128–41. doi: 10.1016/j.jhep.2021.06.023 34171432

[42] Ho RLY Ho IAW. Recent Advances in Glioma Therapy: Combining Vascular Normalization and Immune Checkpoint Blockade. Cancers (Basel) (2021) 13:3686. doi: 10.3390/cancers13153686 34359588 PMC8345045

[43] Xu S Tang L Dai G Luo C Liu Z. Expression of M6a Regulators Correlated With Immune Microenvironment Predicts Therapeutic Efficacy and Prognosis in Gliomas. Front Cell Dev Biol (2020) 8:594112. doi: 10.3389/fcell.2020.594112 33240891 PMC7683617

[44] Lin S Xu H Zhang A Ni Y Xu Y Meng T. Prognosis Analysis and Validation of M(6)A Signature and Tumor Immune Microenvironment in Glioma. Front Oncol (2020) 10:541401. doi: 10.3389/fonc.2020.541401 33123464 PMC7571468

[45] Pan Y Xiao K Li Y Li Y Liu Q. RNA N6-Methyladenosine Regulator-Mediated Methylation Modifications Pattern and Immune Infiltration Features in Glioblastoma. Front Oncol (2021) 11:632934. doi: 10.3389/fonc.2021.632934 33718217 PMC7947873

[46] Du J Ji H Ma S Jin J Mi S Hou K. M6a Regulator-Mediated Methylation Modification Patterns and Characteristics of Immunity and Stemness in Low-Grade Glioma. Brief Bioinform (2021) 22:bbab013. doi: 10.1093/bib/bbab013 33594424

[47] Zheng J Wang X Qiu Y Wang M Yu H Zhou Z. Identification of Critical M(6)A RNA Methylation Regulators With Prognostic Value in Lower-Grade Glioma. BioMed Res Int (2021) 2021:9959212. doi: 10.1155/2021/9959212 34212046 PMC8205593

[48] Arora M Kumari S Singh J Chopra A Chauhan SS. Downregulation of Brain Enriched Type 2 MAGEs Is Associated With Immune Infiltration and Poor Prognosis in Glioma. Front Oncol (2020) 10:573378. doi: 10.3389/fonc.2020.573378 33425727 PMC7787151

[49] Weon JL Yang SW Potts PR. Cytosolic Iron-Sulfur Assembly Is Evolutionarily Tuned by a Cancer-Amplified Ubiquitin Ligase. Mol Cell (2018) 69:113–25.e6. doi: 10.1016/j.molcel.2017.11.010 29225034

[50] Bardhan M Polavarapu K Bevinahalli NN Veeramani PK Anjanappa RM Arunachal G. Megaconial Congenital Muscular Dystrophy Secondary to Novel CHKB Mutations Resemble Atypical Rett Syndrome. J Hum Genet (2021) 66:813–23. doi: 10.1038/s10038-021-00913-1 33712684

[51] Kawashima S Inozume T Kawazu M Ueno T Nagasaki J Tanji E. TIGIT/CD155 Axis Mediates Resistance to Immunotherapy in Patients With Melanoma With the Inflamed Tumor Microenvironment. J Immunother Cancer (2021) 9:e003134. doi: 10.1136/jitc-2021-003134 34795004 PMC8603290

[52] Yang F He Z Duan H Zhang D Li J Yang H. Synergistic Immunotherapy of Glioblastoma by Dual Targeting of IL-6 and CD40. Nat Commun (2021) 12:3424. doi: 10.1038/s41467-021-23832-3 34103524 PMC8187342

