# Supplementary figures and images for: Roles of the m6A Modification of RNA in the Glioblastoma Microenvironment as Revealed by Single-Cell Analyses
Source: Front Immunol. 2022 Apr 26;13:798583. doi: 10.3389/fimmu.2022.798583 (PMC9086907; doi:10.3389/fimmu.2022.798583)

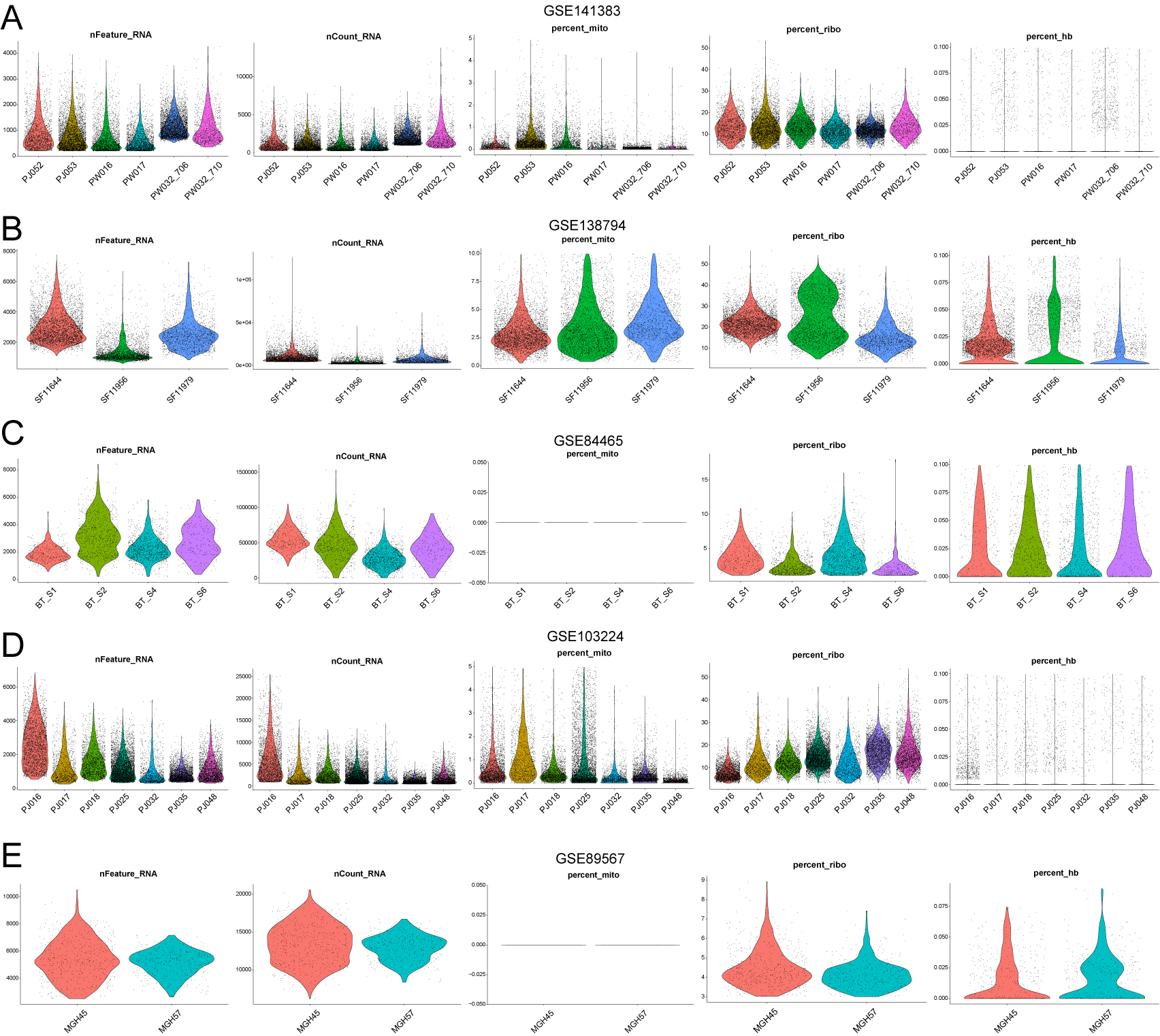

Supplement: Supplementary Figure 1 — Quality control of samples from (A) GSE141383, (B) GSE138794, (C) GSE84465, (D) GSE103224, and (E) GSE89567 datasets. [file Image_1.tif]

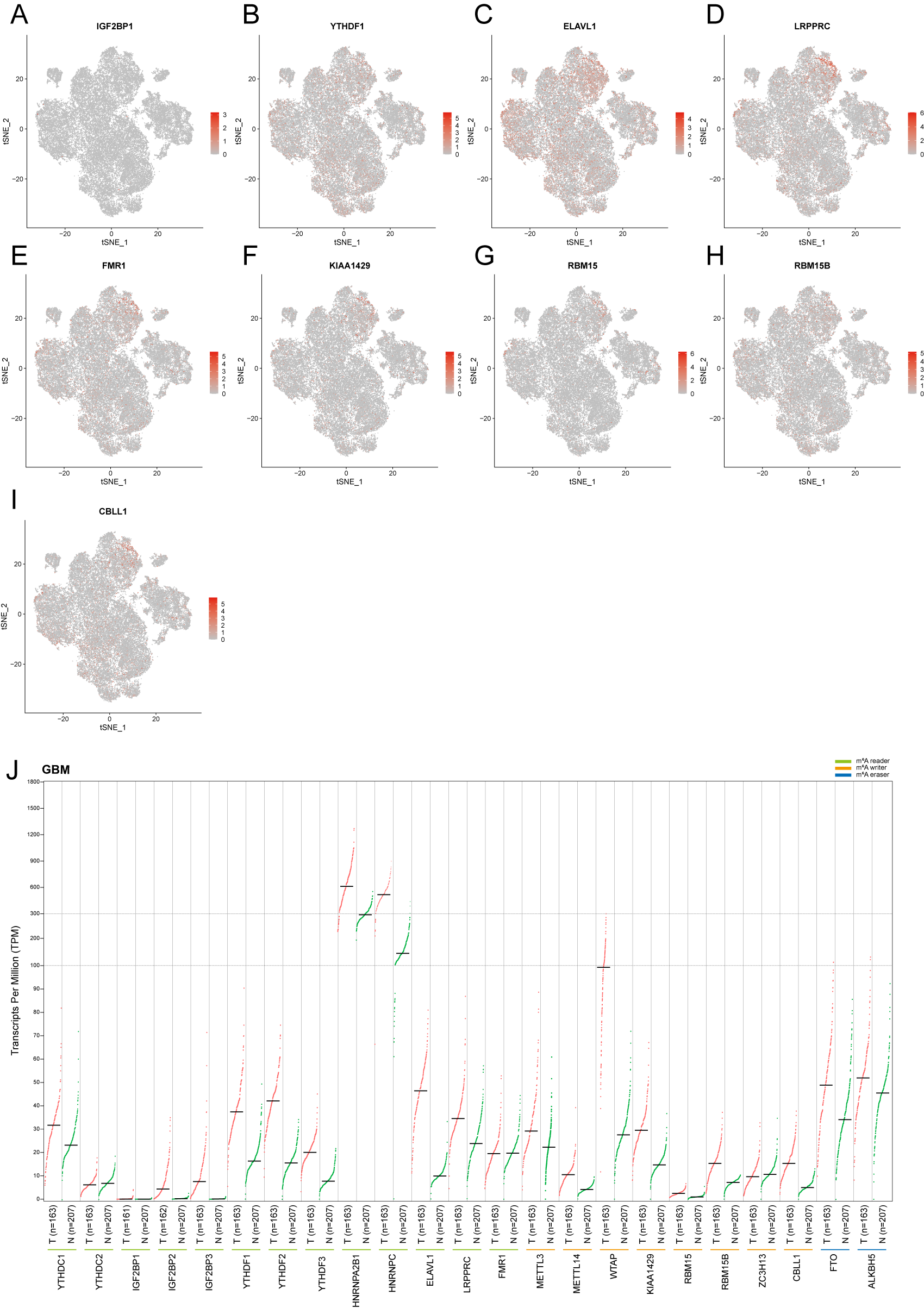

Supplement: Supplementary Figure 2 — Distribution of m6A regulators in GBM microenvironment. (A–I) tSNE plots of 9 m6A regulators. (J) Expression levels of 23 m6A regulators in GBM and normal samples from bulk RNA-seq dataset in GEPIA website. [file Image_2.tif]

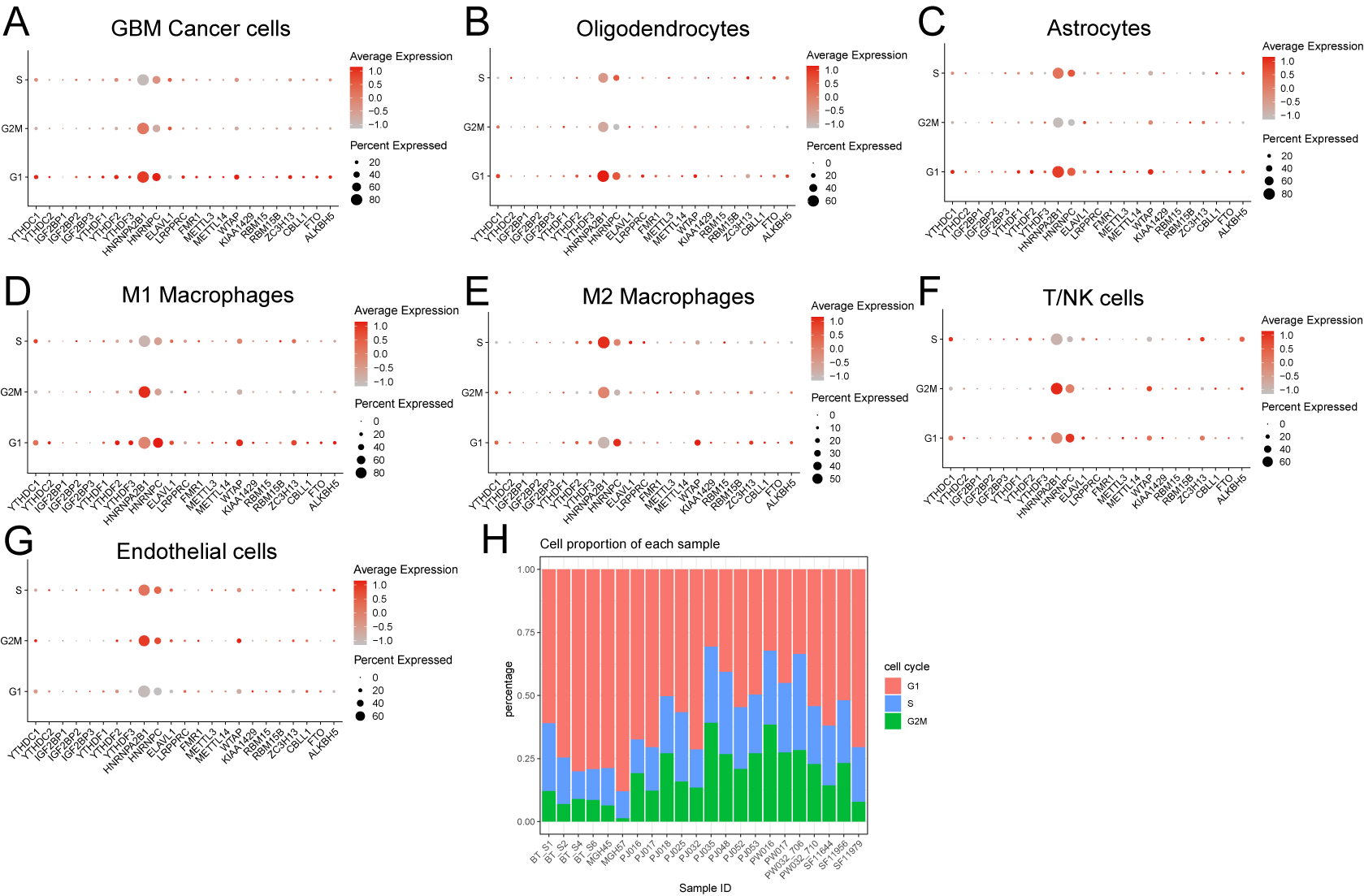

Supplement: Supplementary Figure 3 — Expression levels of m6A regulators in 3 cell cycles. (A-G) Expression levels of m6A regulators in 3 stages of the cell cycle from GBM cancer cells (A), oligodendrocytes (B), astrocytes (C), M1 macrophages (D), M2 macrophages (E), T/NK cells (F), and endothelial cells (G). (H) Distribution of 3 stages of the cell cycle in each included sample. [file Image_3.tif]

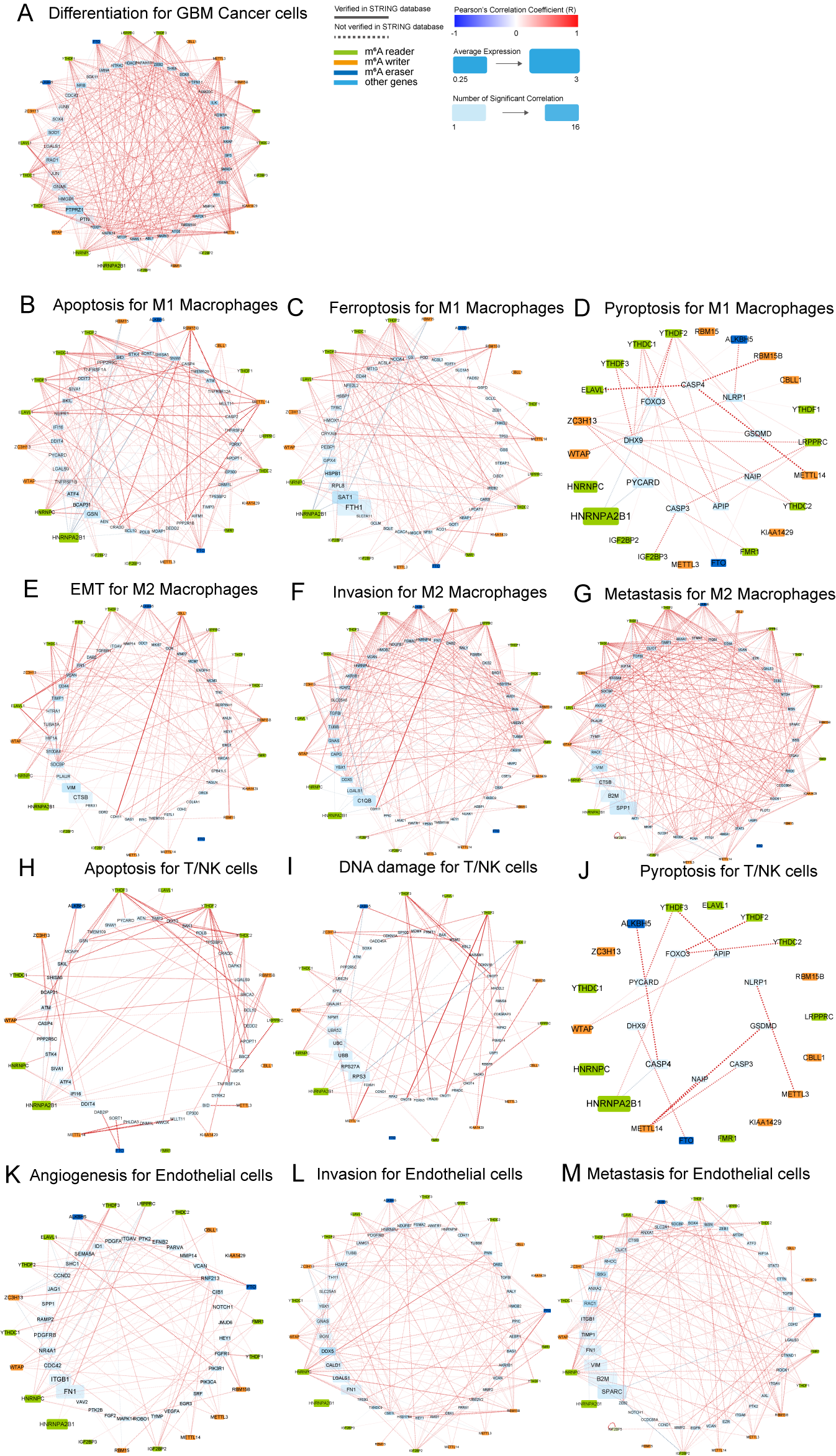

Supplement: Supplementary Figure 4 — correlation network of m6A regulators and functional states. (A) correlation network of m6A regulators and differentiation related genes in GBM cancer cells. (B-D) correlation network of m6A regulators and apoptosis (B), ferroptosis (C), and pyroptosis (D) related genes in M1 macrophages. (E-G) correlation network of m6A regulators and EMT (E), invasion (F), and metastasis (G) related genes in M2 macrophages. (H–J) correlation network of m6A regulators and apoptosis (H), DNA damage (I), and pyroptosis (J) related genes in T/NK cells. (K-M) correlation network of m6A regulators and angiogenesis (K), invasion (L), and metastasis (M) related genes in endothelial cells. [file Image_4.tif]

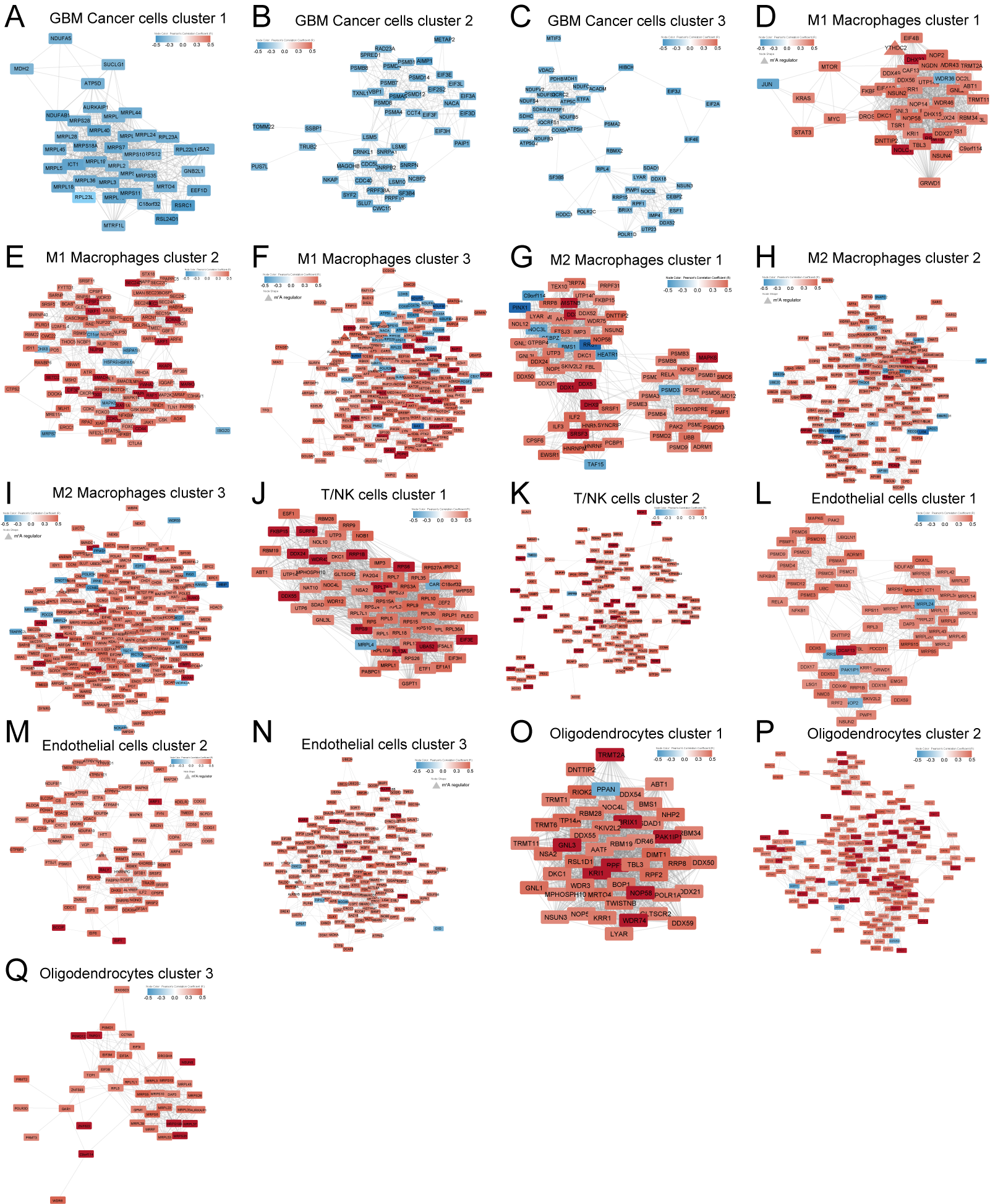

Supplement: Supplementary Figure 5 — m6A related gene clusters and functional annotation. (A-C) PPI networks of cluster 1 (A), cluster 2 (B), and cluster 3 (C) of m6A related genes in GBM cancer cells. (D–F) PPI networks of cluster 1 (D), cluster 2 (E), and cluster 3 (F) of m6A related genes in M1 macrophages. (G-I) PPI networks of cluster 1 (G), cluster 2 (H), and cluster 3 (I) of m6A related genes in M2 macrophages. (J, K) PPI networks of cluster 1 (J), and cluster 2 (K) of m6A related genes in T/NK cells. (L-N) PPI networks of cluster 1 (L), cluster 2 (M), and cluster 3 (N) of m6A related genes in endothelial cells. (O-Q) PPI network and enriched biological process terms of cluster 1 (O), cluster 2 (P), and cluster 3 (Q) of m6A related genes in oligodendrocytes. [file Image_5.tif]

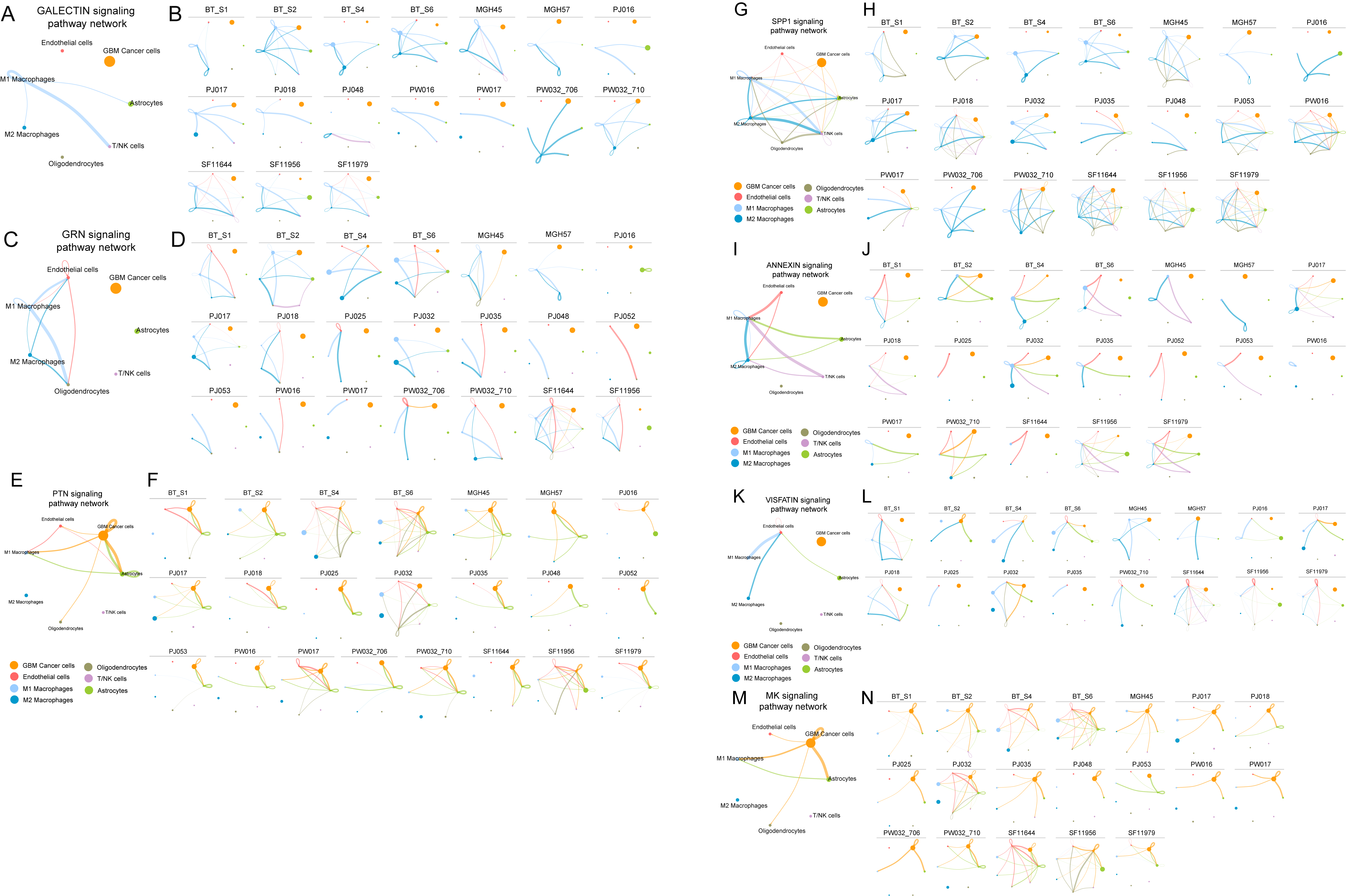

Supplement: Supplementary Figure 6 — Cell communication analysis reveals robust signal pathways in the GBM cancer microenvironment. (A, B) The GALECTIN signaling pathway network as detected in cell communication analyses for all samples (A) and each sample (B). (C, D) The GRN signaling pathway network as detected in cell communication analyses for all samples (C) and each sample (D). (E, F) The PTN signaling pathway network as detected in cell communication analyses for all samples (E) and each sample (F). (G, H) The SPP1 signaling pathway network as detected in cell communication analyses for all samples (G) and each sample (H). (I, J) The ANNEXIN signaling pathway network as detected in cell communication analyses for all samples (I) and each sample (J). (K, L) The VISFATIN signaling pathway network as detected in cell communication analyses for all samples (K) and each sample (L). (M, N) The MK signaling pathway network as detected in cell communication analyses for all samples (M) and each sample (N). [file Image_6.tif]

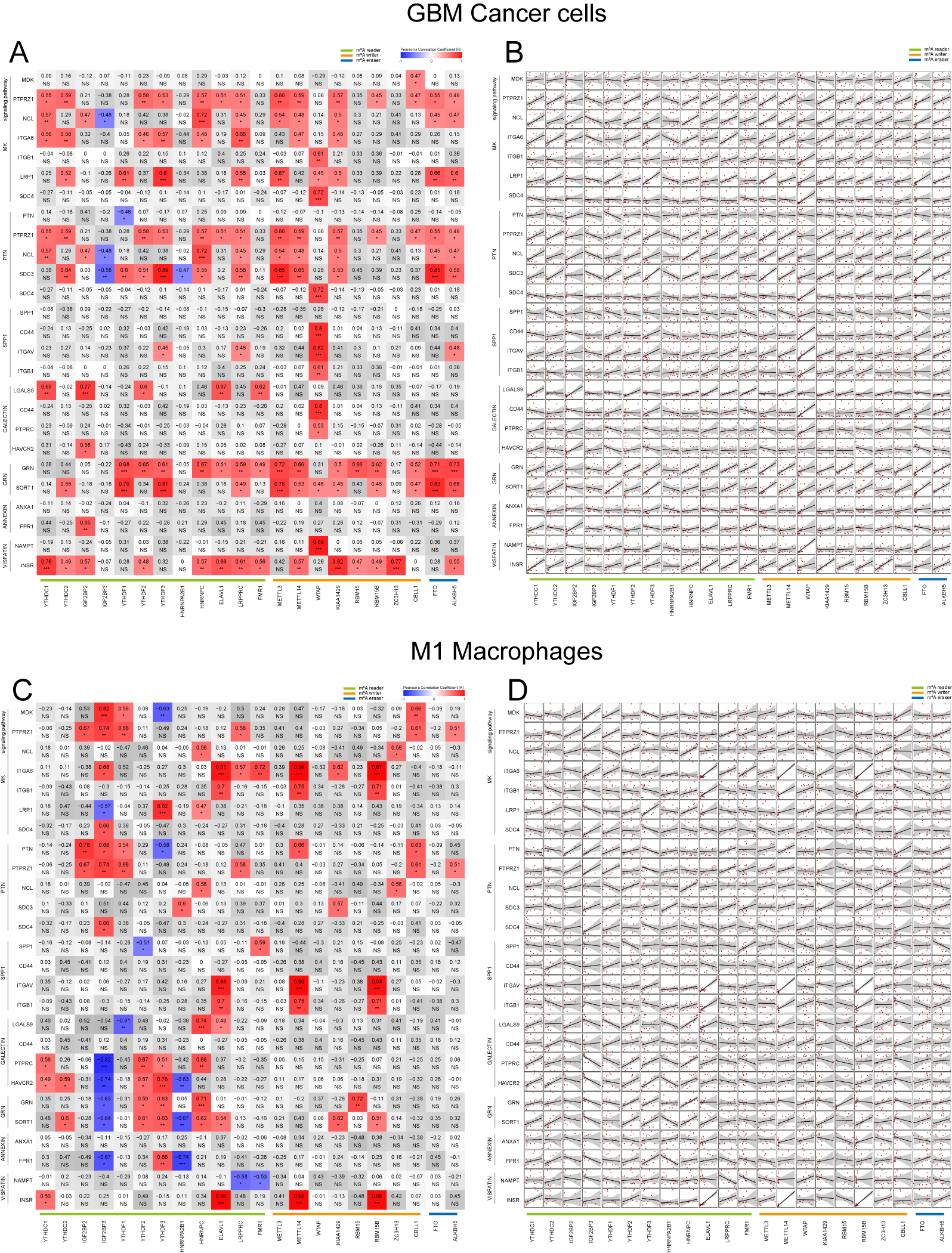

Supplement: Supplementary Figure 7 — Correlation analysis of m6A regulators and genes in signaling pathway networks in GBM cancer cells and M1 macrophages. (A, B) Correlation heatmap (A) and correlation analysis (B) of m6A regulators and genes in signaling pathway networks in GBM cancer cells. (C, D) Correlation heatmap (C) and correlation analysis (D) of m6A regulators and genes in signaling pathway networks in M1 macrophages. NS: P > 0.05, *P ≤ 0.05, **P ≤ 0.01, ***P ≤ 0.001. [file Image_7.tif]

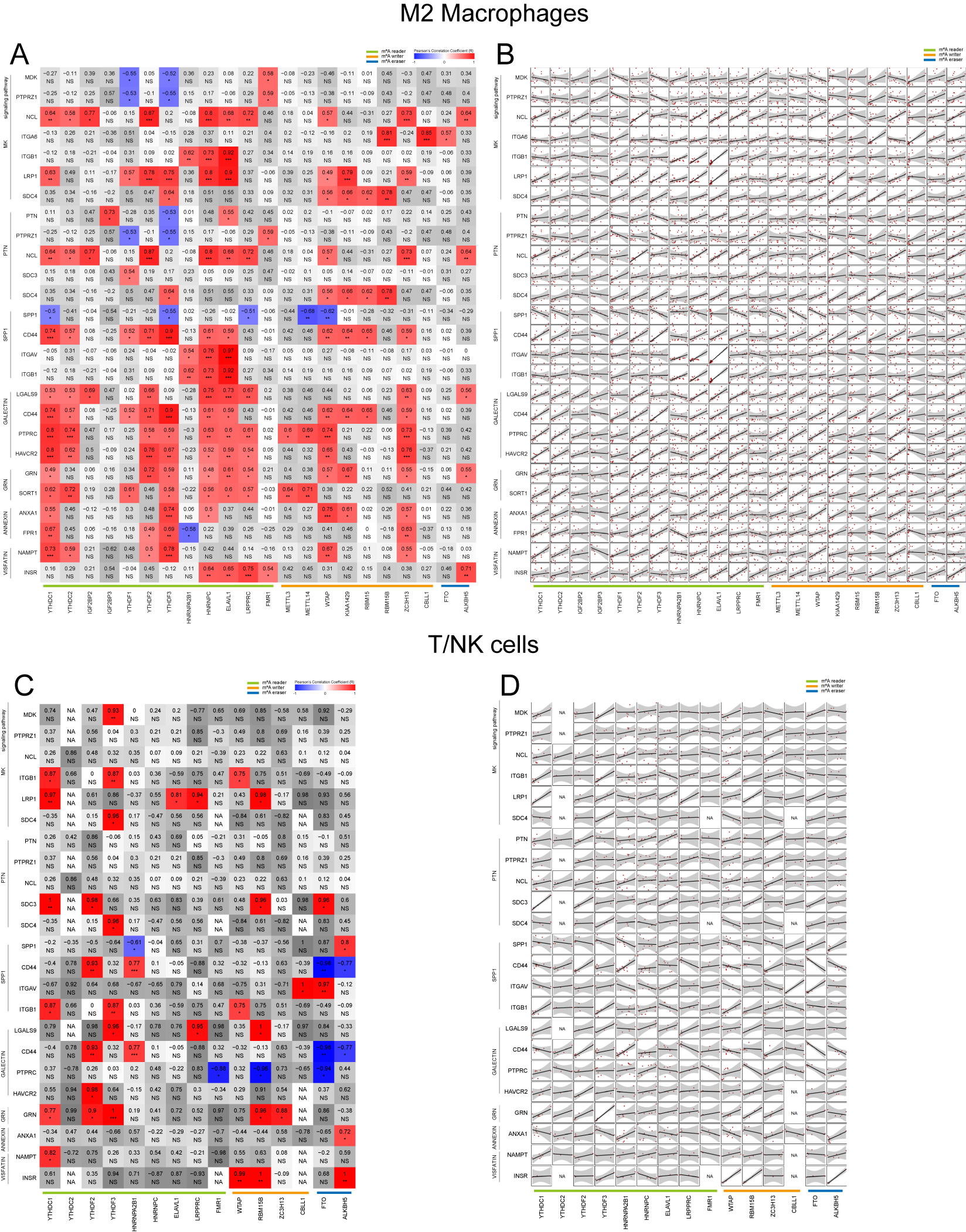

Supplement: Supplementary Figure 8 — Correlation analysis of m6A regulators and genes in signaling pathway networks in M2 macrophages and T/NK cells. (A, B) Correlation heatmap (A) and correlation analysis (B) of m6A regulators and genes in signaling pathway networks in M2 macrophages. (C, D) Correlation heatmap (C) and correlation analysis (D) of m6A regulators and genes in signaling pathway networks in T/NK cells. NS: P > 0.05, *P ≤ 0.05, **P ≤ 0.01, ***P ≤ 0.001. [file Image_8.tif]

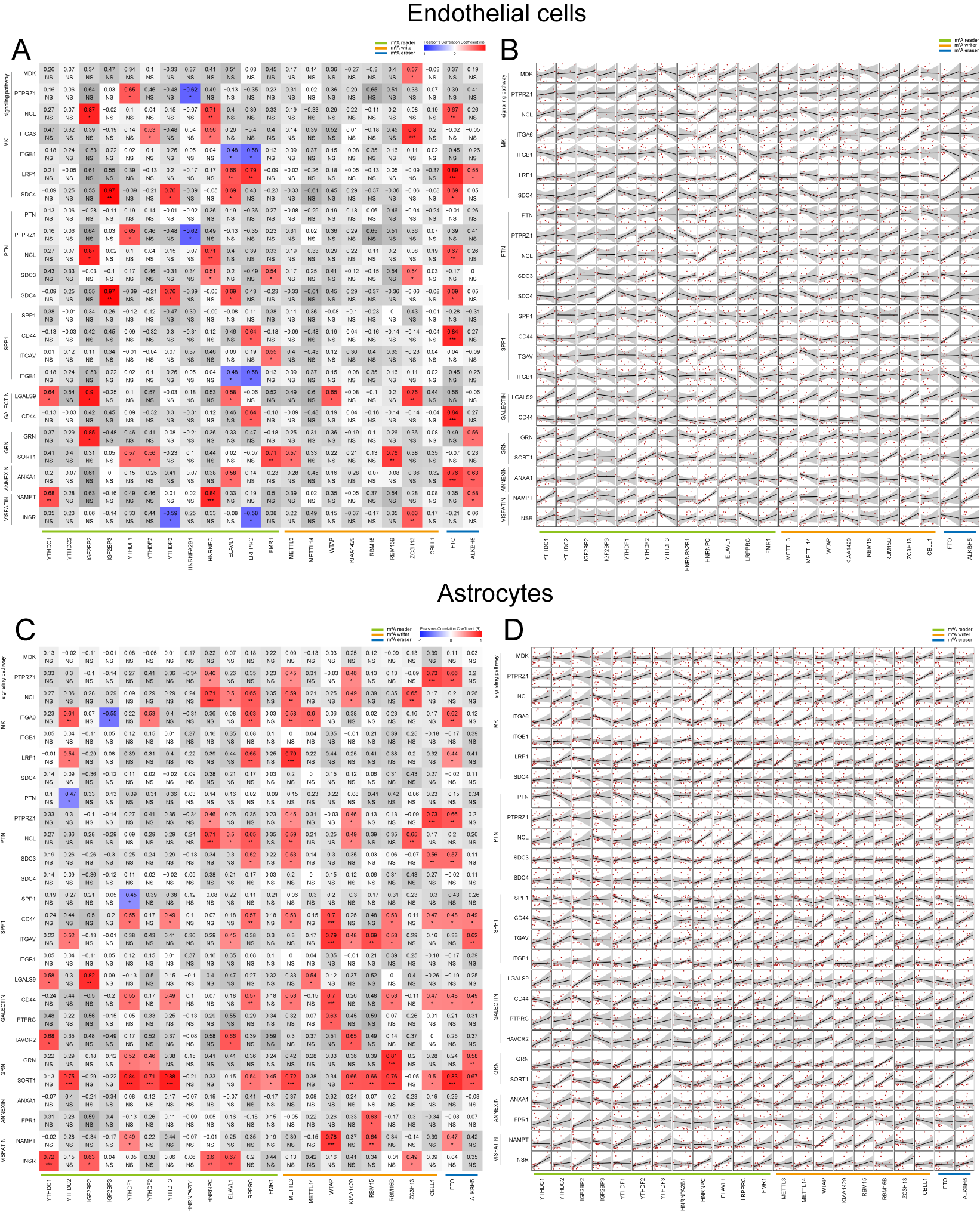

Supplement: Supplementary Figure 9 — Correlation analysis of m6A regulators and genes in signaling pathway networks in endothelial cells and astrocytes. (A, B) Correlation heatmap (A) and correlation analysis (B) of m6A regulators and genes in signaling pathway networks in endothelial cells. (C, D) Correlation heatmap (C) and correlation analysis (D) of m6A regulators and genes in signaling pathway networks in astrocytes. NS: P > 0.05, *P ≤ 0.05, **P ≤ 0.01, ***P ≤ 0.001. [file Image_9.tif]

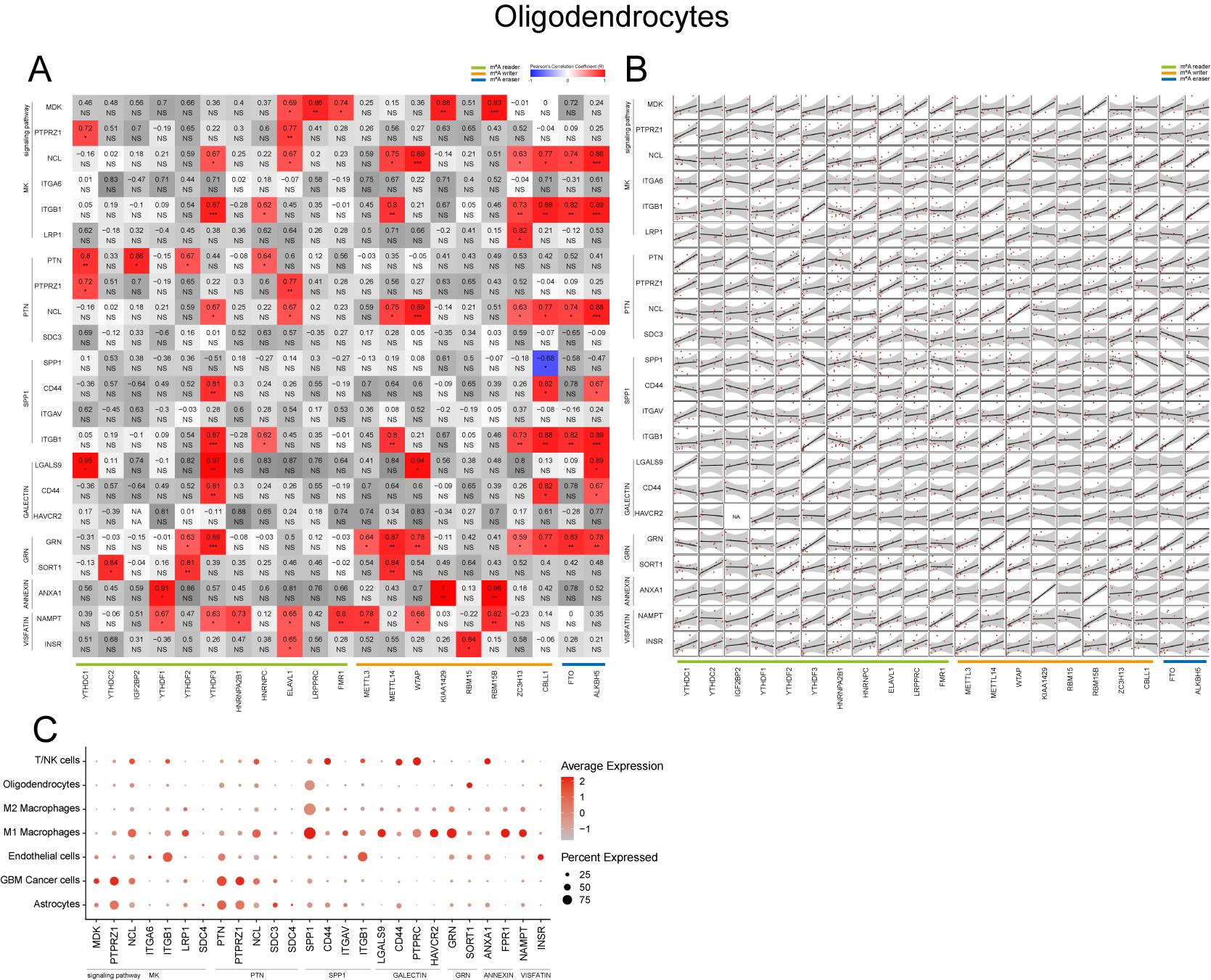

Supplement: Supplementary Figure 10 — Correlation analysis of m6A regulators and genes in signaling pathway networks in oligodendrocytes. (A, B) Correlation heatmap (A) and correlation analysis (B) of m6A regulators and genes in signaling pathway networks in oligodendrocytes. (C) Expression levels of genes in signaling pathway networks in 7 cell types. NS: P > 0.05, *P ≤ 0.05, **P ≤ 0.01, ***P ≤ 0.001. [file Image_10.tif]

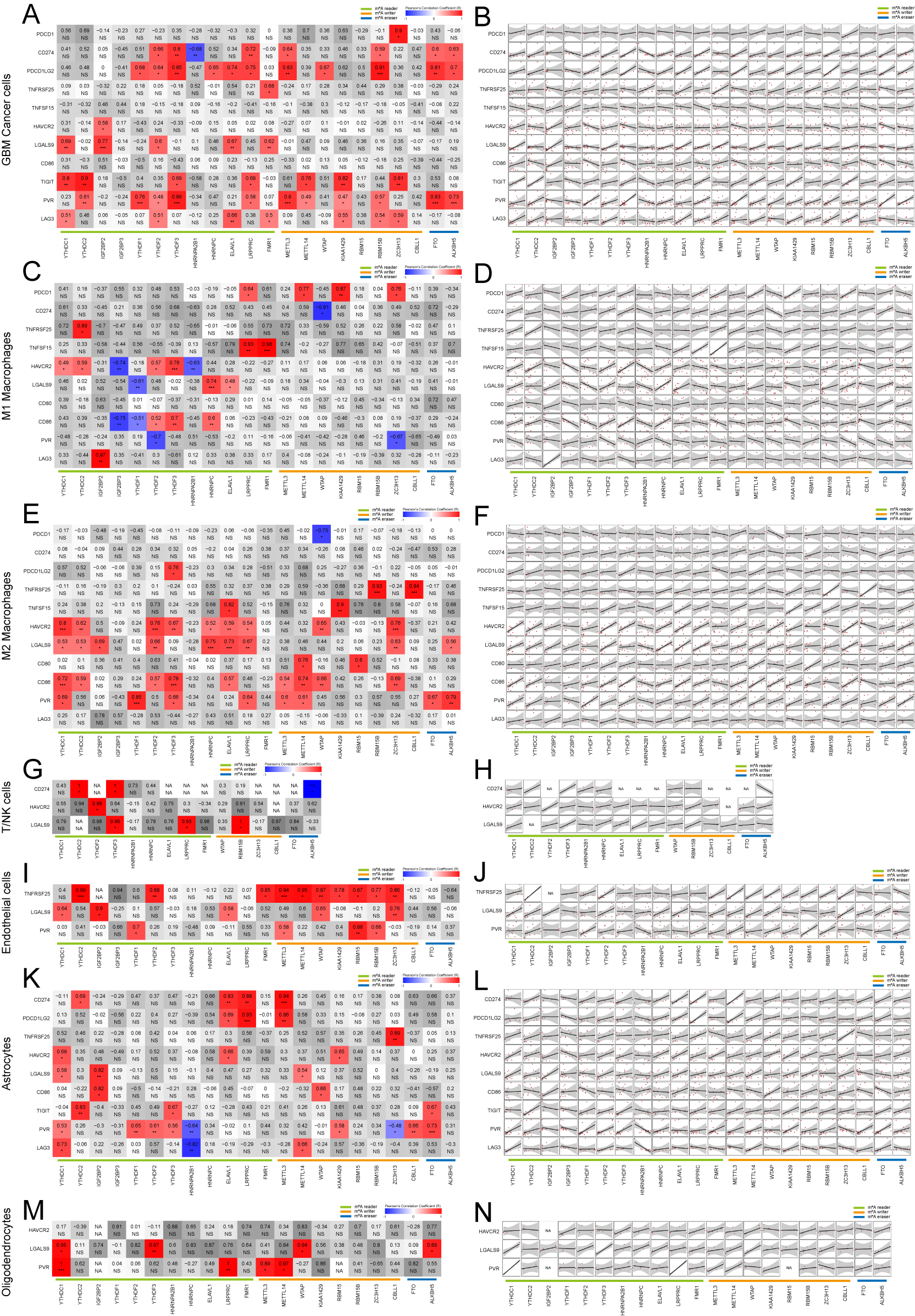

Supplement: Supplementary Figure 11 — Correlation analysis of m6A regulators and immune checkpoints (ICPs). (A, B) Correlation heatmap (A) and correlation analysis (B) of m6A regulators and ICPs in GBM cancer cells. (C, D) Correlation heatmap (C) and correlation analysis (D) of m6A regulators and ICPs in M1 macrophages. (E, F) Correlation heatmap (E) and correlation analysis (F) of m6A regulators and ICPs in M2 macrophages. (G, H) Correlation heatmap (G) and correlation analysis (H) of m6A regulators and ICPs in T/NK cells. (I, J) Correlation heatmap (I) and correlation analysis (J) of m6A regulators and ICPs in endothelial cells. (K, L) Correlation heatmap (K) and correlation analysis (L) of m6A regulators and ICPs in astrocytes. (M, N) Correlation heatmap (M) and correlation analysis (N) of m6A regulators and ICPs in oligodendrocytes. NS: P > 0.05, *P ≤ 0.05, **P ≤ 0.01, ***P ≤ 0.001. [file Image_11.tif]

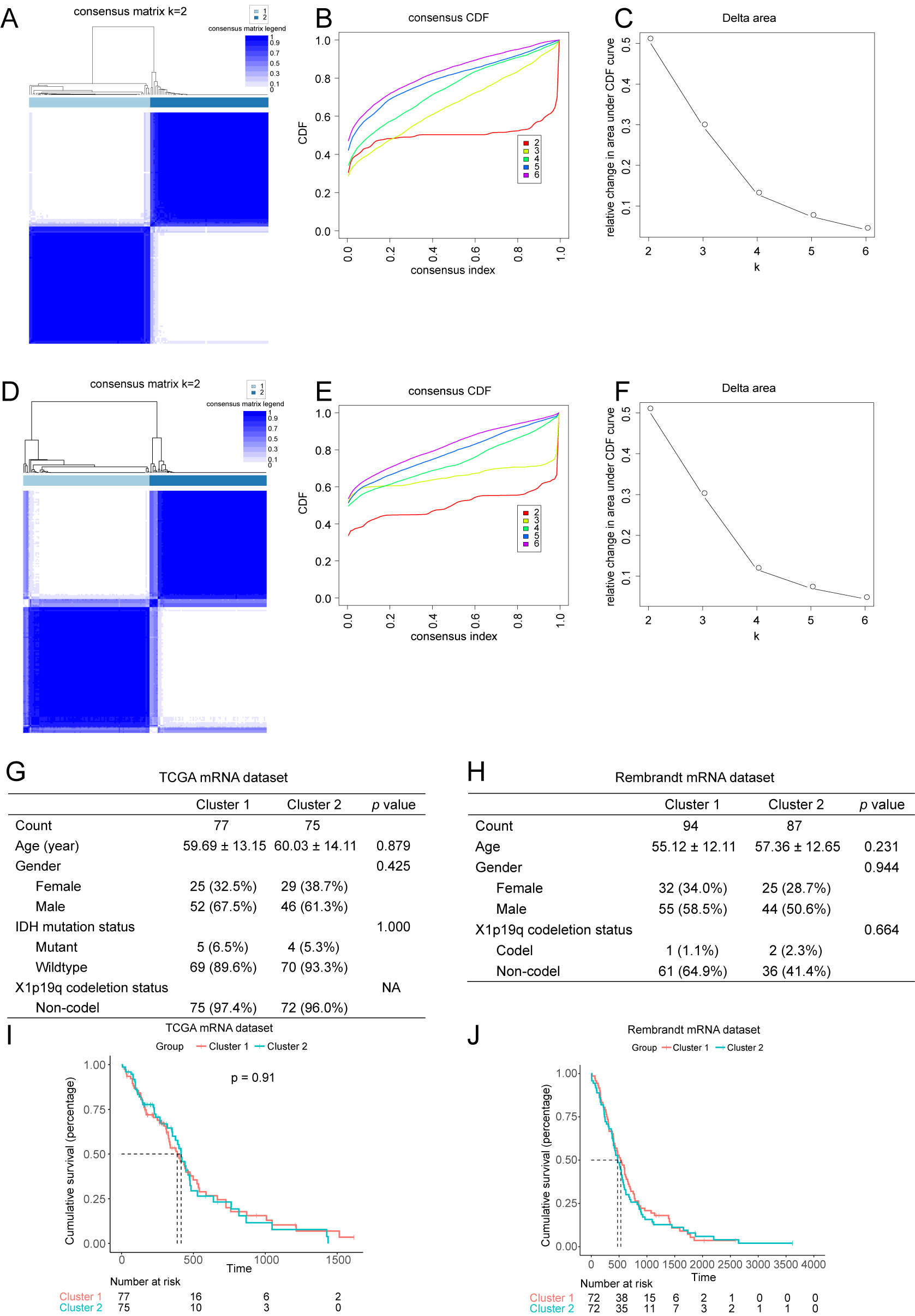

Supplement: Supplementary Figure 12 — Identification and validation of 2 clusters of GBM patients in bulk RNA-seq analysis. (A, D) Consensus clustering matrix for k = 2, which was the optimal cluster number in the TCGA dataset (A) and REMBRANDT dataset (D). (B, E) CDF curves of the consensus score (k = 2-6) in the TCGA dataset (B) and REMBRANDT dataset (E). (C, F) Relative change in the area under the CDF curve (k = 2-6) in the TCGA dataset (C) and REMBRANDT dataset (F). (G, H) Clinical features of 2 identified clusters in the TCGA dataset (G) and REMBRANDT dataset (H). (I, J) Kaplan-Meier survival analyses of 2 identified clusters in the TCGA dataset (I) and REMBRANDT dataset (J) [file Image_12.tif]

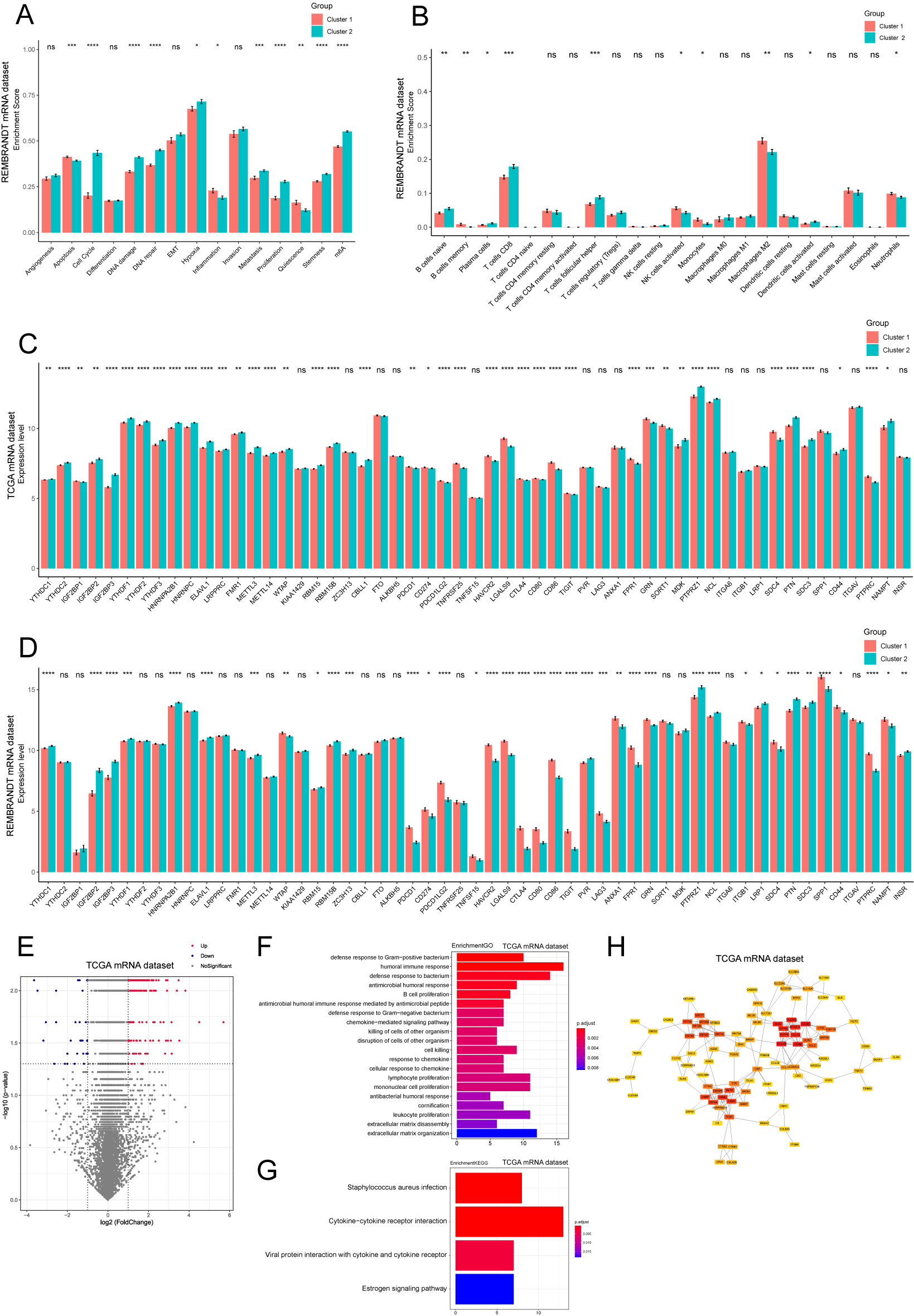

Supplement: Supplementary Figure 13 — comparison of 2 identified clusters of GBM patients in bulk RNA-seq analysis. (A) Bar charts illustrating the differences of functional state scores between 2 identified clusters for REMBRANDT dataset. (B) Bar charts illustrating the differences of functional state scores between 2 identified clusters for REMBRANDT dataset. (C, D) Bar charts illustrating the differences of expression levels of m6A regulators, immune checkpoints, and genes in detected cell communication signaling pathway networks between 2 identified clusters for TCGA (C) and REMBRANDT (D) dataset. (E) Volcano plot of differentially expressed genes (DEGs) between 2 identified clusters in TCGA dataset. (F, G) GO (F) and KEGG (G) analyses of up-regulated DEGs in TCGA dataset. (H) PPI network showing hub genes of up-regulated DEGs in TCGA dataset. ns: P > 0.05, *P ≤ 0.05, **P ≤ 0.01, ***P ≤ 0.001, ****P ≤ 0.0001. [file Image_13.tif]

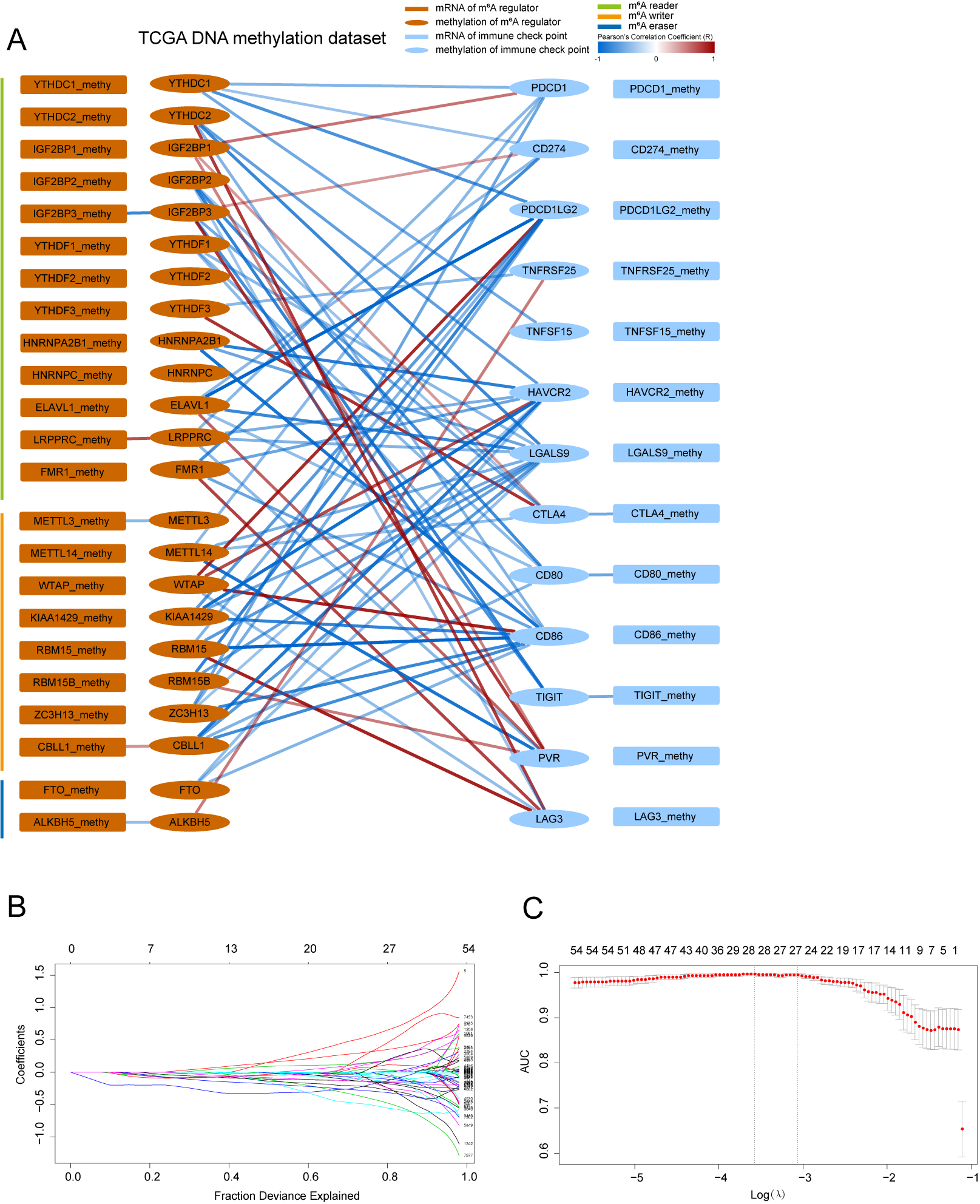

Supplement: Supplementary Figure 14 — Multiomic analysis and LASSO analysis. (A) Correlation network of the expression levels and methylation levels of m6A regulators and ICPs in all GBM samples from TCGA dataset. (B, C) LASSO regression analysis of training dataset (TCGA). [file Image_14.tif]

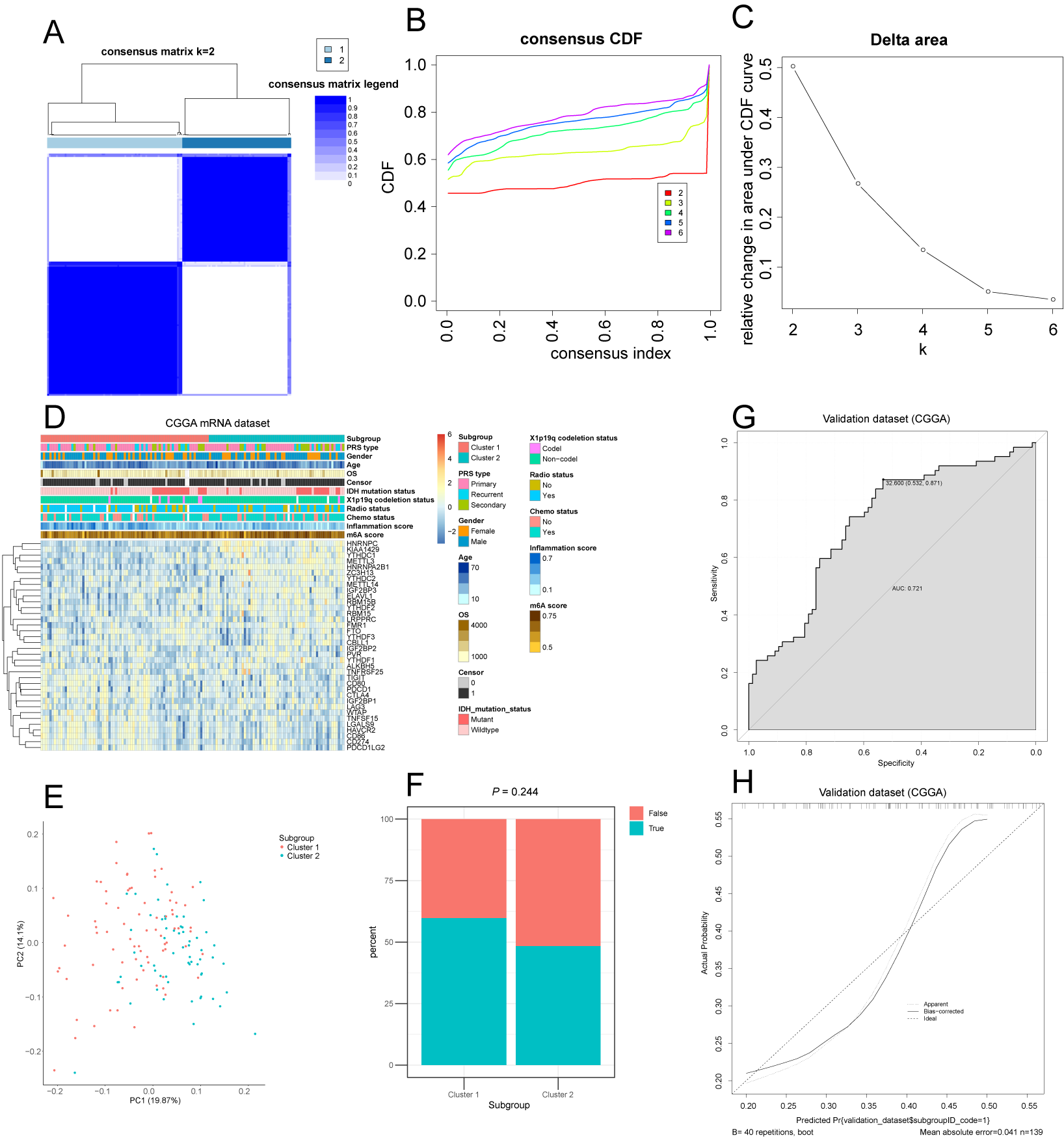

Supplement: Supplementary Figure 15 — Bulk RNA-seq analysis with CGGA dataset. (A), Consensus clustering matrix for k = 2, which was the optimal cluster number in the CGGA dataset. (B) CDF curves of the consensus score (k = 2-6) in the CGGA dataset. (C) Relative change in the area under the CDF curve (k = 2-6) in the CGGA dataset. (D) Heatmap of m6A regulators and immune checkpoints (ICPs) revealed the different m6A-ICP expression patterns for 2 clusters identified by consensus clustering for CGGA dataset. (E) Principal component analysis of 2 identified clusters for CGGA dataset. (F) Predicted potential therapeutic response of ICP inhibitors of 2 identified clusters for CGGA dataset. (G) ROC curve of the nomogram distinguishing 2 identified clusters for validation dataset (CGGA). (H) Calibrate plot of the nomogram distinguishing 2 identified clusters for validation dataset (CGGA). [file Image_15.tif]
